# Single-atom catalysts-based catalytic ROS clearance for efficient psoriasis treatment and relapse prevention via restoring ESR1

**DOI:** 10.1038/s41467-023-42477-y

**Published:** 2023-10-25

**Authors:** Xiangyu Lu, Le Kuai, Fang Huang, Jingsi Jiang, Jiankun Song, Yiqiong Liu, Si Chen, Lijie Mao, Wei Peng, Ying Luo, Yongyong Li, Haiqing Dong, Bin Li, Jianlin Shi

**Affiliations:** 1 Shanghai Tenth People’s Hospital, Shanghai Frontiers Science Center of Nanocatalytic Medicine, Clinical Center For Brain And Spinal Cord Research, School of Medicine, Tongji University, Shanghai, 200092 China; 2 Shanghai Institute of Ceramics, Chinese Academy of Sciences, Research Unit of Nanocatalytic Medicine in Specific Therapy for Serious Disease, Chinese Academy of Medical Sciences, Shanghai, 200050 China; 3 Department of Dermatology, Yueyang Hospital of Integrated Traditional Chinese and Western Medicine, Shanghai University of Traditional Chinese Medicine, Shanghai, 200437 China; 4 Institute of Dermatology, Shanghai Academy of Traditional Chinese Medicine, Shanghai, 201203 China; 5 Key Laboratory of Spine and Spinal Cord Injury Repair and Regeneration, Ministry of Education, Tongji Hospital, School of Medicine, Tongji University, Shanghai, 200065 China; 6 Shanghai Skin Disease Hospital, School of Medicine, Tongji University, Shanghai, 200443 China; 7 Institute of Waste Treatment and Reclamation, College of Environment Science and Engineering, Tongji University, Shanghai, 200092 China

**Keywords:** Psoriasis, Bioinspired materials, Biomedical materials

## Abstract

Psoriasis is a common inflammatory disease of especially high recurrence rate (90%) which is suffered by approximately 3% of the world population. The overexpression of reactive oxygen species (ROS) plays a critical role in psoriasis progress. Here we show that biomimetic iron single-atom catalysts (FeN_4_O_2_-SACs) with broad-spectrum ROS scavenging capability can be used for psoriasis treatment and relapse prevention via related gene restoration. FeN_4_O_2_-SACs demonstrate attractive multiple enzyme-mimicking activities based on atomically dispersed Fe active structures, which are analogous to those of natural antioxidant enzymes, iron superoxide dismutase, human erythrocyte catalase, and ascorbate peroxidase. Further, in vitro and in vivo experiments show that FeN_4_O_2_-SACs can effectively ameliorate psoriasis-like symptoms and prevent the relapse with augmented efficacy compared with the clinical drug calcipotriol. Mechanistically, estrogen receptor 1 (ESR1) is identified as the core protein upregulated in psoriasis treatment through RNA sequencing and bioinformatic analysis. Together, this study provides a proof of concept of psoriasis catalytic therapy (PCT) and multienzyme-inspired bionics (MIB).

## Abstract (web summary)

Psoriasis is a common inflammatory disease, and the overproduction of reactive oxygen species (ROS) in skin lesions plays a critical role in the progress of psoriasis. Here, the authors report the use of multienzyme-inspired biomimetic iron single-atom catalysts (FeN_4_O_2_-SACs) with broad-spectrum ROS-scavenging capability for psoriasis treatment and relapse prevention via related gene restoration.

## Introduction

Psoriasis is an inflammatory skin disorder featuring chronic, relapsing erythematous plaques covered with silvery-white scurfy scales^1–3^. The prevalence rate of psoriasis ranges from 0.47% to 6.6% in China, America, Europe, and Australia, and continues to rise^4^. Sustained inflammation and non-regulated keratinocyte proliferation, especially the burst releases of inflammatory factors in the psoriasis development, are the primary culprits involved in the pathogenesis^5,6^. Although current therapies permit the control of psoriatic inflammation, the prolonged immunosuppression, however, increases body’s susceptibility to infection and skin cancer^7–9^. Additionally, psoriasis is often accompanied by other metabolic diseases, such as cardiovascular disease and diabetes, which will cause serious harms to human health. More seriously, the biggest clinical challenge for psoriasis treatment is symptom recurrence after drug discontinuation (approximately 90%)^10^. Thus, alternative therapeutic modalities for psoriasis inflammation alleviation and relapse intervention are urgently needed^11–14^.

Reactive oxygen species (ROS), such as superoxide anions (O_2_•^-^) and hydrogen peroxide (H_2_O_2_), are modulators of inflammation that can stimulate inflammatory cytokine release, thereby stimulating the proliferation of keratinocytes^15–17^. Excessive ROS accumulation caused by oxidative/anti-oxidative imbalance and resultantly elevated oxidative damage are observed in psoriatic patients^12^. From this perspective, downregulating the ROS level at the lesion site would be highly desirable in the treatment of psoriasis. Nevertheless, ROS cannot be reduced sustainably by consuming antioxidants such as bilirubin^18,19^ and monomethyl fumarate^20^. Further investigation has revealed that in response to ROS, the body has a natural anti-oxidation system, mainly composed of antioxidant enzymes, such as catalase (CAT) and superoxide dismutase (SOD), which scavenge O_2_•^-^ and H_2_O_2_ through catalytic reactions to maintain homeostasis^21–23^. Unfortunately, it has been discovered that these antioxidant enzymes are underexpressed in patients suffering psoriasis, which could prevent efficient ROS scavenging and even exacerbate the symptom^24^. Thus, one potential psoriasis treatment strategy is to restore redox homeostasis at the lesion by utilizing the catalytic reactions of antioxidant enzymes, thereby ameliorating inflammatory infiltration and hyperproliferation of HaCaT cells, i.e., to achieve psoriasis catalytic therapy (PCT). Nevertheless, the natural antioxidant enzymes are particularly problematic in clinical applications (e.g., single catalytic activity, low permeability, and high cost). One effective strategy is to design biomimetic catalytic materials with multiple catalytic activities by simulating the pivotal structural features of natural enzymes, which is herein defined as multienzyme-inspired bionics (MIB)^25–30^.

Fortunately, single-atom catalysts (SACs) with tunable active metal centers and coordination environments provide an effective solution in mimicking the highly evolved catalytic center of natural enzymes at the atomic level^31–33^. However, most previous work has focused on mimicking the active center of a single natural enzyme. To better construct antioxidant enzyme-like catalysts, this work explores a strategy to simultaneously simulate the catalytic centers of multiple natural antioxidant enzymes.

**Fig. 1:**
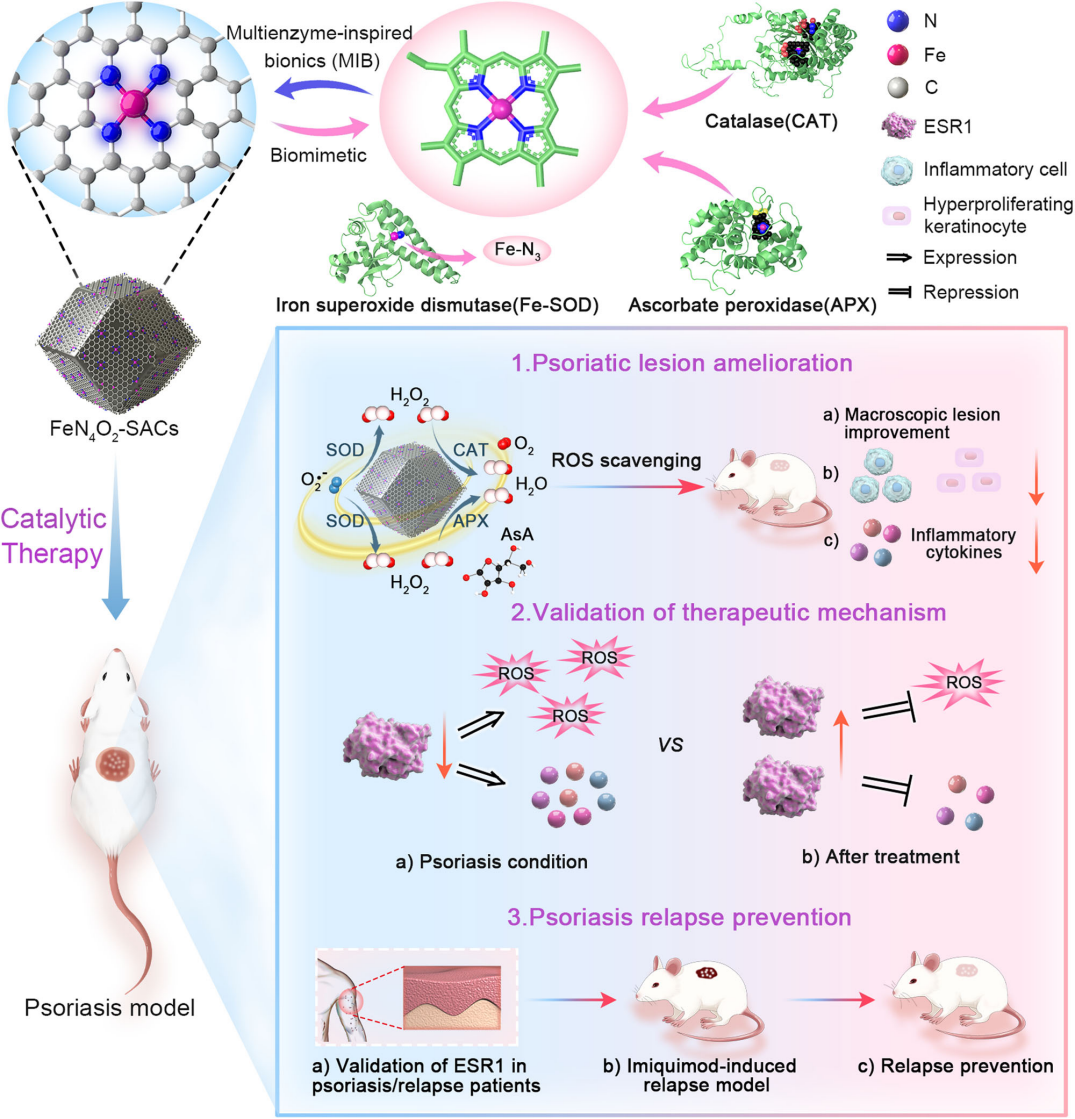
Schematic illustration of FeN_4_O_2_-SACs for psoriasis catalytic therapy. Inspired by the common structure feature for the activity centers of three antioxidant enzymes, FeN_4_O_2_-SACs with SOD-, CAT-, and APX-like activities are synthesized for ROS scavenging in psoriasis treatment. As expected, FeN_4_O_2_-SACs effectively ameliorate psoriatic skin lesions in vitro and in vivo. The core of the action of FeN_4_O_2_-SACs is further validated to be the upregulation of ESR1, which is downregulated in psoriasis and relapse patients. Finally, the effective prevention of relapse is achieved by FeN_4_O_2_-SACs.

In the antioxidant enzymes human erythrocyte CAT (PDB: 1dgf)^34^ and ascorbate peroxidase (APX; PDB: 1v0h)^35^, the catalytic centers are the Fe-porphyrin (Heme) structures, in which the active Fe is linked with four nitrogen atoms. Interestingly, Fe-SOD (PDB: 1avm)^36^ features FeN_3_ as an active site (Fig. 1). Consequently, we hypothesize that Fe-based SACs including Fe active site coordinated by nitrogen atoms could potentially act as multi-active natural antioxidant enzyme mimics.

In this work, we formulate a biomimetic Fe-N_4_O_2_ structure-based, atomically dispersed Fe-N-C catalyst (FeN_4_O_2_-SACs) to efficiently scavenge different types of ROS by its multiple CAT, APX, and SOD-mimicking activities. Due to their effectiveness in downregulating inflammatory factor levels, preventing keratinocyte proliferation and ROS generation, FeN_4_O_2_-SACs present significant efficacy in treating psoriasiform dermatitis, and notably, reduce the relapse of psoriasis after drug discontinuation. Furthermore, the underlying mechanism of the catalytic therapy for psoriasis is the up-regulation of estrogen receptor 1 (ESR1) in the FeN_4_O_2_-SACs treatment, which is largely reduced in the clinical samples of psoriasis and relapse patients. Taken together, this biomimetic catalyst delivers a “safe, effective, and long-term” therapeutic modality for psoriasis treatment as a typical example of multienzyme-inspired bionics (Fig. 1).

## Results

### Synthesis of FeN_4_O_2_-SACs

**Fig. 2:**
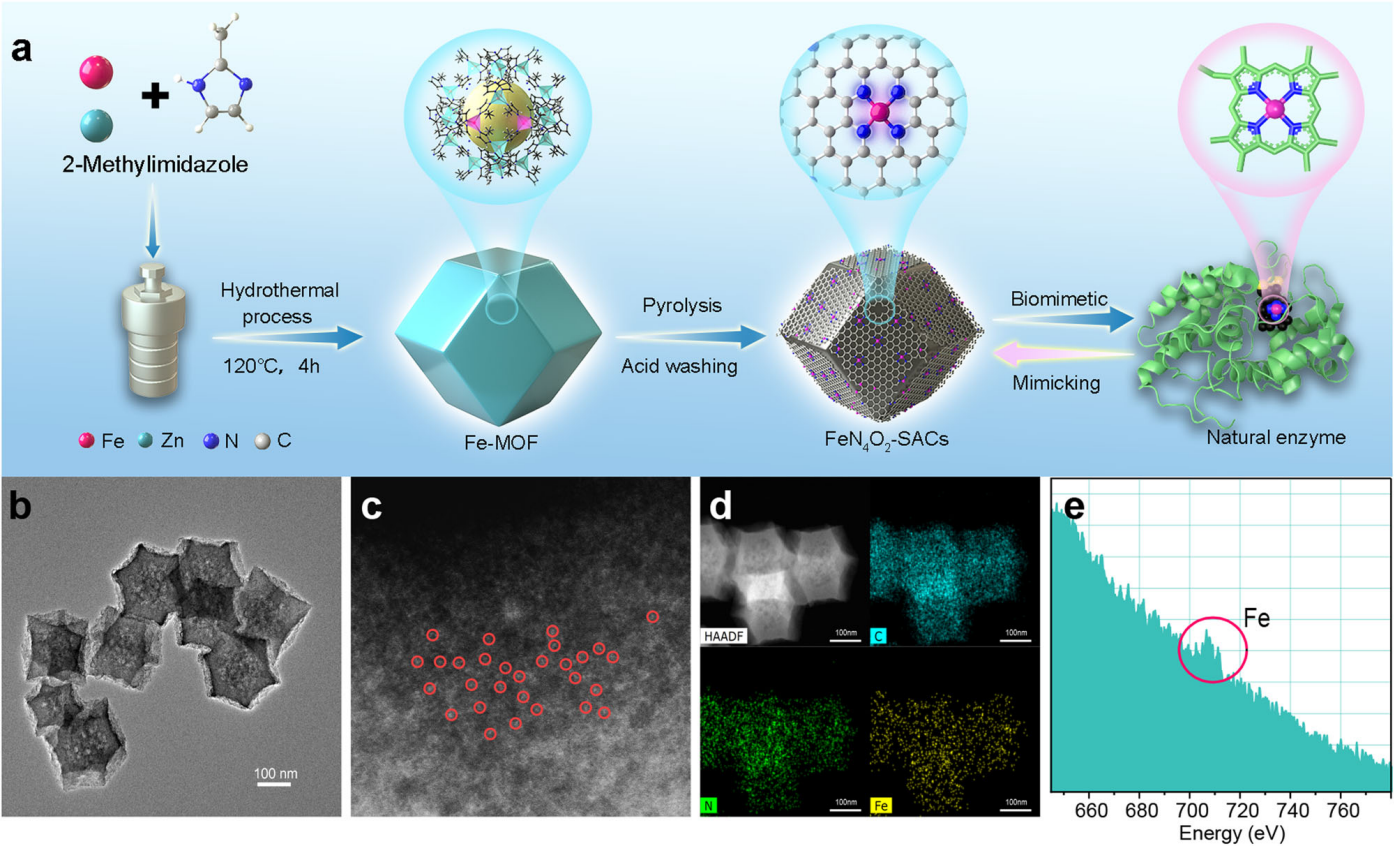
Synthesis and characterization of FeN_4_O_2_-SACs. **a** Synthetic process of biomimetic FeN_4_O_2_-SACs. **b** TEM image of FeN_4_O_2_-SACs. *n* = 3 samples with similar results. **c** AC HAADF-STEM image of FeN_4_O_2_-SACs, with single iron atoms marked using red circles. *n* = 3 samples with similar results. **d** HAADF-STEM image and corresponding EDX mappings of FeN_4_O_2_-SACs: C (blue), N (green), Fe (yellow). **e** EELS atomic spectrum of Fe elements in FeN_4_O_2_-SACs. The element-specific absorption edge is highlighted by a red circle. Source data are provided as a Source Data file.

A schematic illustration of the synthesis of FeN_4_O_2_-SACs is provided in Fig. 2a. Briefly, Fe-doped ZIF-8 (Fe-MOF) was pyrolysed at 800°C for 3 h and then, the pyrolysate was leached with sulfuric acid to obtain FeN_4_O_2_-SACs. Analogously, N-doped porous carbon (NC) without Fe doping was prepared as a control.

Transmission electron microscopy (TEM) images show that both Fe-MOF and FeN_4_O_2_-SACs are relatively uniform in size with cubic morphologies (Supplementary Fig. 1 and Fig. 2b). Scanning electron microscopy (SEM) further displays the dodecahedral structure of FeN_4_O_2_-SACs (Supplementary Fig. 2). Notably, the sizes of FeN_4_O_2_-SACs (≈150 nm) particles are relatively smaller than those of Fe-MOF, and a pore structure appears on the surface of FeN_4_O_2_-SACs, which originated from the evaporation and overflow of Zn species during pyrolysis^37^. Surface area analysis demonstrates that FeN_4_O_2_-SACs enrich with micro- and mesopores with large specific surface area (Supplementary Fig. 3), which facilitate the exposure of catalytically active sites. The individually scattered bright dots in the aberration-corrected high-angle annular dark-field scanning TEM (AC HAADF-STEM) image confirm the atomic dispersion of Fe atoms (Fig. 2c). In parallel, the electron energy loss spectrum (EELS) and X-ray photoelectron spectroscopy (XPS) identify the presence of Fe signals (Fig. 2e and Supplementary Fig. 4), demonstrating successful Fe doping. The concentration of Fe is 0.43% as quantified by inductively coupled plasma optical emission spectrometry (ICP-OES). The HAADF-STEM image and energy-dispersive spectrum (EDS) further indicate uniform distributions of C, N, and Fe elements (Fig. 2d). To determine whether Fe-derived crystalline structures were present or not, high-resolution TEM (HRTEM) and selected area electron diffraction (SAED) were performed. As depicted in Supplementary Figs. 5 and 6, no metal crystals formed in FeN_4_O_2_-SACs, which is consistent with X-ray diffractometer (XRD) pattern (Supplementary Fig. 7 and Supplementary Discussion). Overall, we have successfully synthesized FeN_4_O_2_-SACs with isolated Fe atoms dispersed in the carbon matrix, homogeneous size, and porous structure.

### Structure of FeN_4_O_2_-SACs

**Fig. 3:**
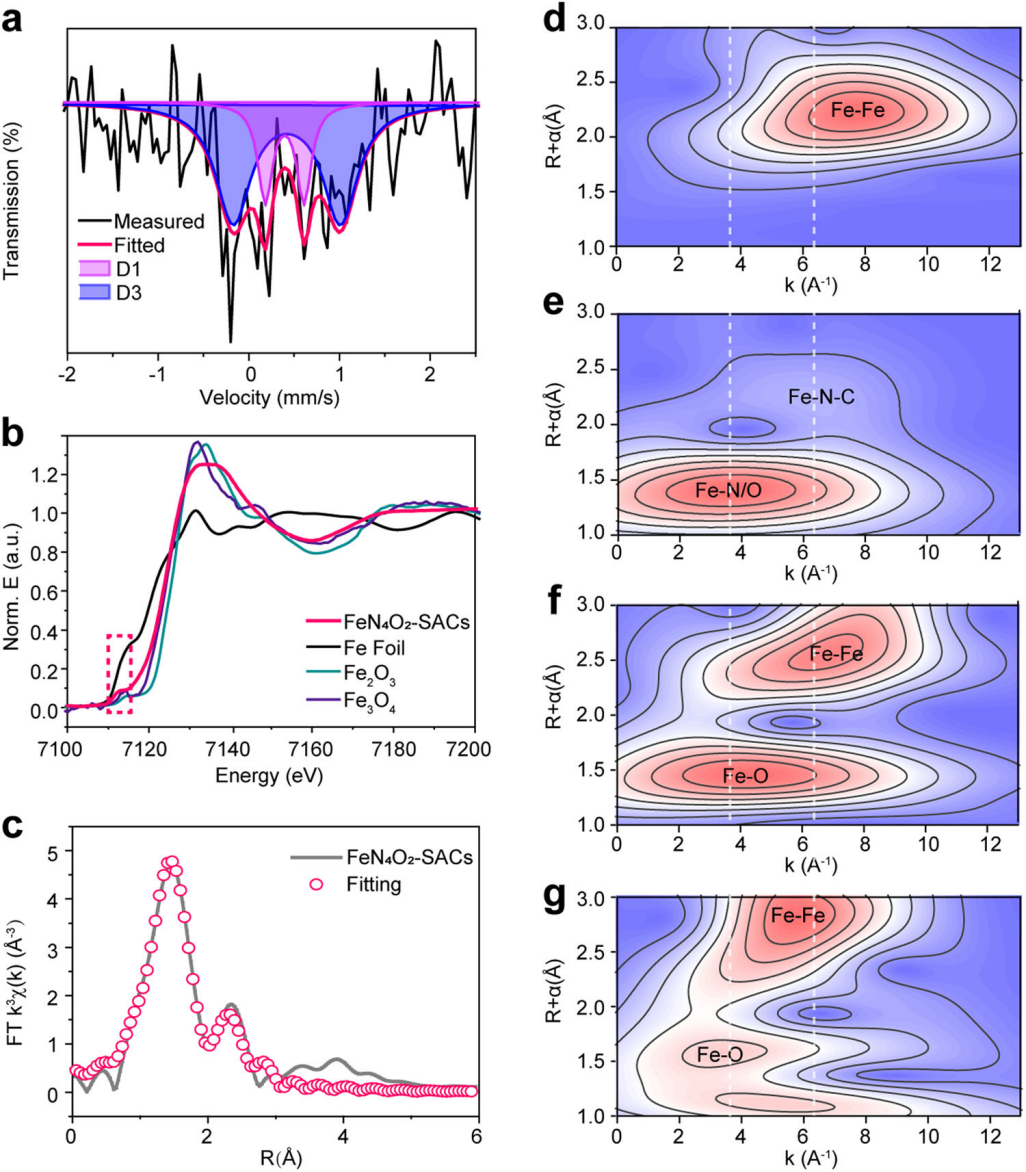
Structure of FeN_4_O_2_-SACs. **a**^57^ Fe Mössbauer spectrum. **b** Normalized XANES spectra of FeN_4_O_2_-SACs and reference samples (Fe Foil, Fe_2_O_3_, Fe_3_O_4_). **c** EXAFS fitting in R space. **d–g** Wavelet transformation of Fe Foil (**d**), FeN_4_O_2_-SACs (**e**), Fe_2_O_3_ (**f**), and Fe_3_O_4_ (**g**). Source data are provided as a Source Data file.

The structure of FeN_4_O_2_-SACs was further investigated by various techniques. Fourier transform infrared (FTIR) spectra and Raman spectra show that FeN_4_O_2_-SACs possess a carbon structure similar to that of NC, which might be related to the low level of Fe doping (Supplementary Figs. 8 and 9 and Supplementary Discussion). Next, Mössbauer spectroscopy was used to determine the electronic state, coordination, and electron spin configuration of FeN_4_O_2_-SACs^38^. Figure 3a shows that FeN_4_O_2_-SACs display the well-documented D1 and D3 doublets attributable to Fe-N species, based on the isomer shift (δ_iso_) and quadrupole splitting (ΔE_Q_) values (Supplementary Table 1). The parameters of the first doublet D1 and third doublet D3 were assigned to the Fe^2+^N_4_ centers and low spin Fe^3+^N_4_ centers (heme-like), respectively^39,40^. The relative absorption area (74.4%) of D3 illustrates the significant presence of heme moieties in FeN_4_O_2_-SACs. In addition, Fe in FeN_4_O_2_-SACs is biased towards the 3+ valence state, which favors antioxidant reactions rather than peroxidase-like catalytic reactions^41,42^. Notably, no significant Fe clusters are evident in the Mössbauer spectrum.

XAFS, a powerful technique for atomic level structure characterization, was carried out to identify the chemical state and coordination environment of the Fe species^43^. Normalized X-ray absorption near-edge structure (XANES) curves verify that the pre-edge position of FeN_4_O_2_-SACs matches that of Fe_3_O_4_, indicating that the Fe valency in FeN_4_O_2_-SACs falls in between +2 and +3, similar to the results of the Mössbauer spectrum (Fig. 3b). The corresponding Fourier-transformed extended X-ray absorption fine structure (FT-EXAFS) spectra (Fig. 3c) reveal a dominant peak at 1.47 Å, which could be designated as the Fe-N(O) scattering path in the first shell. Meanwhile, a weak peak at 2.31 Å is observed in FeN_4_O_2_-SACs, which can be ascribed as Fe-N-C in the second shell^44,45^. Similar results are evidenced by the wavelet transform (WT) plot (Fig. 3d–g). By comparison with reference samples of Fe Foil, Fe_2_O_3_ and Fe_3_O_4_, FeN_4_O_2_-SACs do not display the intensity maximum corresponding to Fe-Fe, proving that all the Fe species are present in the single-atom state. Then, least-square EXFAS fitting was performed to obtain quantitative structural parameters. As shown in Supplementary Fig. 10 and Supplementary Table 2, the Fe atoms in FeN_4_O_2_-SACs exhibit a six-fold coordination by N/O atoms and a mean Fe-N/O bond length of 1.99 Å. Since adjacent coordination elements in the periodic table (such as N and O) could not fit well in EXAFS, the presence of Fe-O coordination shells could not be excluded^44^. It was speculated that this might be the structure of Fe-N_4_O_2_ in combination with Mössbauer spectrum results and the literature^46^; and the oxygen element could derive from the atmospheric oxygen in the experiment, though slight amount of Fe-N_5_ or Fe-N_6_ could be present^44,46^. XPS further shows that the N types coordinated with the Fe atoms are pyridine N, pyrrole N, and graphene N, corresponding to 398.6, 399.8, and 401.2 eV, respectively (Supplementary Fig. 11)^47^. Altogether, FeN_4_O_2_-SACs share a similar Fe-N_4_ structure to heme, which has encouraged us to further investigate its enzymatic activity.

### SOD, CAT, and APX-like catalytic activities of FeN_4_O_2_-SACs

**Fig. 4:**
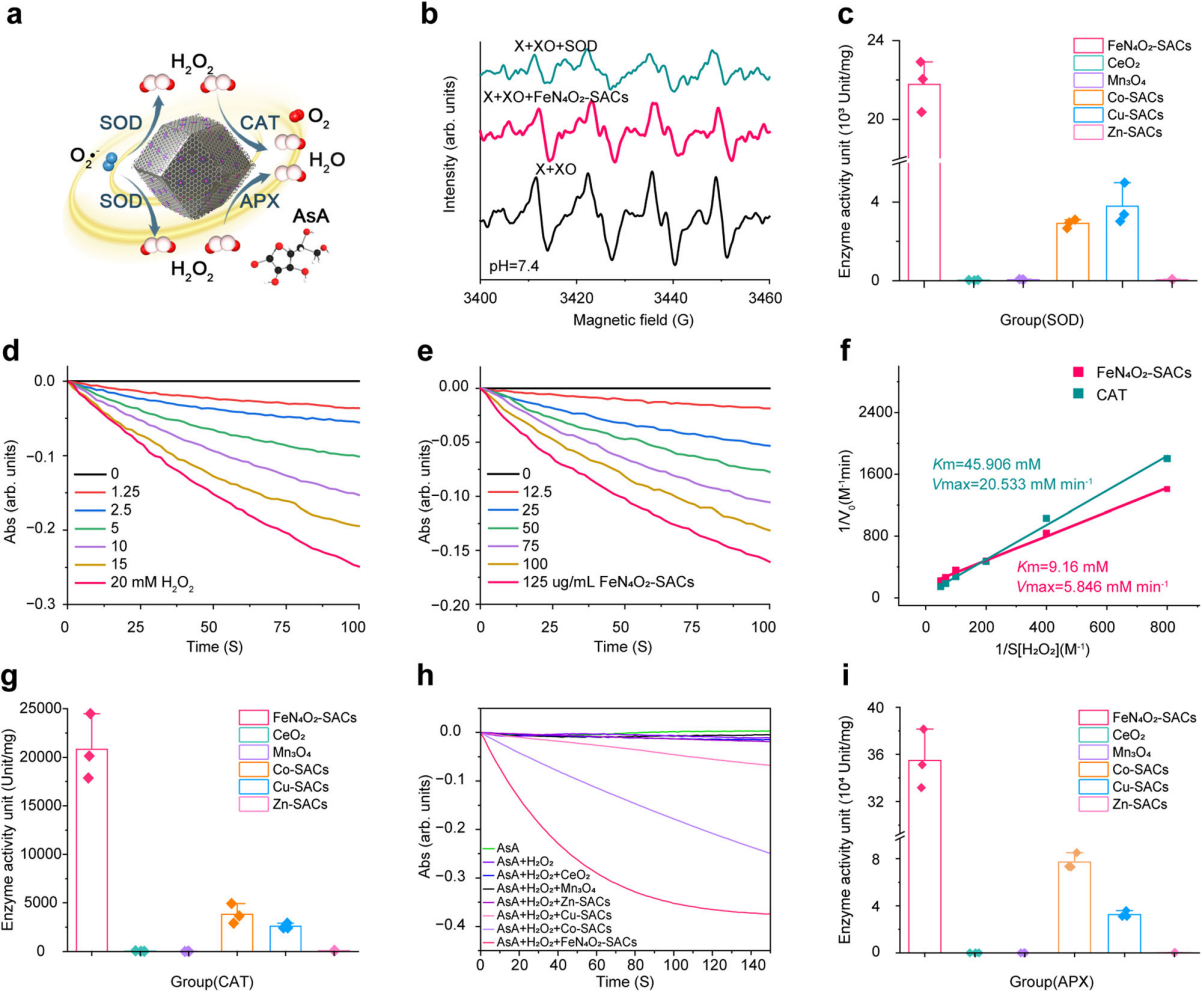
CAT, SOD, and APX-like activities of FeN_4_O_2_-SACs. **a** Schematic illustration of ROS clearance by FeN_4_O_2_-SACs. **b** ESR spectra of FeN_4_O_2_-SACs and natural SOD for O_2_•^-^ clearance at pH=7.4 (X: xanthine; XO: xanthine oxidase). **c** Comparison of SOD-like activities between FeN_4_O_2_-SACs and control materials CeO_2_, Mn_3_O_4_, Co-SACs, Cu-SACs and Zn-SACs by WST-8 colorimetric analysis. *n* = 3 independent experiments and data are presented as mean ± SD. **d** CAT-like assay for eliminating varied concentrations of H_2_O_2_ using 125 μg/ml FeN_4_O_2_-SACs by UV absorption tests. **e** CAT-like assay for eliminating 10 mM H_2_O_2_ using FeN_4_O_2_-SACs of varied concentrations. **f** Lineweaver‒Burk plots for FeN_4_O_2_-SACs and natural CAT at varied concentrations of H_2_O_2_ (0-20 mM). **g** Comparison of the CAT-like activities among FeN_4_O_2_-SACs and control materials Mn_3_O_4_, CeO_2_, Co-SACs, Cu-SACs and Zn-SACs. *n* = 3 independent experiments and data are presented as mean ± SD. **h** AsA characteristic absorption intensity decreases upon the additions of H_2_O_2_ of different samples. **i** Comparison of the APX-like activities between FeN_4_O_2_-SACs and other materials Mn_3_O_4_ and CeO_2_, Co-SACs, Cu-SACs and Zn-SACs. *n* = 3 independent experiments and data are presented as mean ± SD. Source data are provided as a Source Data file.

O_2_•^-^ is a kind of ROS with strong oxidizing and destructive potentials. As a pivotal O_2_•^-^ scavenger, the enzyme SOD catalyses the disproportionation of O_2_•^-^ into H_2_O_2_ and O_2_ (Fig. 4a)^48^. Here, the clearance of O_2_•^-^ was verified via electron spin resonance (ESR) measurements using 5-tert-butoxycarbonyl 5-methyl-1-pyrroline N-oxide (BMPO) as the O_2_•^-^ trapping reagent. ESR spectra show that O_2_•^-^ is consumed efficiently by FeN_4_O_2_-SACs at pH 7.4 with the characteristic peak intensity similar to that in the presence of natural SOD, indicating that FeN_4_O_2_-SACs have the SOD-like activity (Fig. 4b)^49^. Likewise, this result has been demonstrated at pH 6 and 8 (Supplementary Fig. 12). Next, the SOD-like performance was quantified with a representative WST-8 colorimetric analysis. As shown in Supplementary Figs. 13 and 14, the O_2_•^-^ scavenging rates of FeN_4_O_2_-SACs and natural SOD increase with increasing concentrations, and the inhibition rate of 0.13 μg/ml Fe in FeN_4_O_2_-SACs is almost equivalent to 0.49 μg/ml natural SOD. Finally, to evaluate the O_2_•^-^ scavenging efficiency of FeN_4_O_2_-SACs, the commercial anti-inflammatory nanozymes (Mn_3_O_4_ and CeO_2_) and metal single-atom catalysts (Co-SACs, Cu-SACs, Zn-SACs) were employed as controls^50–53^. The results show that FeN_4_O_2_-SACs exhibit the highest SOD-like activity, and the activities of Fe in FeN_4_O_2_-SACs are 622, 342, and 377 times those of Ce in CeO_2_, Mn in Mn_3_O_4_, and Zn in Zn-SACs, respectively (Fig. 4c, Supplementary Table 3).

Although SOD can protect the organism from O_2_•^-^ damage, H_2_O_2_ generated via SOD can destroy DNA, proteins and lipids^54,55^. Clearance of H_2_O_2_ from the body relies heavily on the enzyme CAT, which catalyzes the conversion of H_2_O_2_ to H_2_O and O_2_ (Fig. 4a)^56–58^. To assess the CAT-like activity of FeN_4_O_2_-SACs, changes in the UV absorbance for H_2_O_2_ at 240 nm were measured initially. Fig. 4d, e shows clear time- and concentration-dependent decreases of the absorbance of H_2_O_2_ at fixed concentration of FeN_4_O_2_-SACs but varied concentrations of H_2_O_2_, or vice versa. A similar result is obtained when FeN_4_O_2_-SACs are replaced by natural CAT (Supplementary Fig. 15). We next quantified the elimination rate of H_2_O_2_. From Supplementary Fig. 16, the elimination rate of H_2_O_2_ by 0.54 μg/ml Fe in FeN_4_O_2_-SACs is comparable to that of 62.5 μg/ml natural CAT. Additionally, the Michaelis constant (*K*_m_) and maximum reaction velocity (*V*_m_) are calculated from Lineweaver–Burk plots (Fig. 4f). The *K*_m_ values of FeN_4_O_2_-SACs and natural CAT are 9.16 and 45.906 mM, respectively, and the *V*_m_ values are 5.856 and 20.533 mM/min, respectively, which suggest that the natural CAT exhibits higher catalytic activity, whereas FeN_4_O_2_-SACs possess stronger affinity to H_2_O_2_. Subsequently, the effects of pH on FeN_4_O_2_-SACs and natural CAT activity were scrutinized. Supplementary Fig. 17 demonstrates that the enzymatic activities of both FeN_4_O_2_-SACs and natural CAT increase with increasing pH. More interestingly, FeN_4_O_2_-SACs show higher activity than natural CAT in the presence of excess base (pH > 9) because H_2_O_2_/O_2_ has a relatively low redox potential under alkaline condition and is more readily oxidized by Fe^3+^(ref. ^59^). Then, the CAT-like performance of FeN_4_O_2_-SACs was compared with those of CeO_2_ and Mn_3_O_4_. At the same mass concentration, FeN_4_O_2_-SACs markedly outperform the others in promoting the decomposition of H_2_O_2_ and producing transparent bubbles (Supplementary Fig. 18). The enzyme activity was further quantified with a CAT assay kit. It is apparent from Fig. 4g that FeN_4_O_2_-SACs present the best CAT-like activity, followed by Mn_3_O_4_ and CeO_2_. The calculated enzyme activity of Fe in FeN_4_O_2_-SACs is respectively 2121 and 471 times that of Ce in CeO_2_ and Mn in Mn_3_O_4_. Finally, the catalytic activity of other commercially available single-atom catalysts in the decomposition of H_2_O_2_ was tested, further confirming the highest catalytic performance of FeN_4_O_2_-SACs in comparison with several reported nanozymes (Fig. 4g). Collectively, the above results demonstrate that FeN_4_O_2_-SACs possess excellent CAT-like enzymatic activity.

In addition, natural APX also catalyses the dissociation of H_2_O_2_ into H_2_O using ascorbic acid (AsA) as the particular electron donor (Fig. 4a). Here, the APX-like activity was characterized by monitoring the absorbance of AsA at 290 nm. As Fig. 4h, i illustrates, FeN_4_O_2_-SACs exhibit a much higher activity in promoting the elimination of AsA than Co-SACs and Cu-SACs in the presence of H_2_O_2_, while Mn_3_O_4_, CeO_2_ and Zn-SACs hardly exhibit such activities. The calculated AsA elimination activities of various materials are listed in Supplementary Table 3.

In summary, we have demonstrated that FeN_4_O_2_-SACs manifest a homogeneous, amorphous, dodecahedral structure with single Fe-N_4_O_2_ active sites being embedded in the structure, which endows the materials with remarkable or significant SOD-, CAT-, and APX-like activities. Such activities are greatly higher than those of typical anti-inflammatory nanozymes (Mn_3_O_4_ and CeO_2_) and commercial Co-SACs, Cu-SACs, and Zn-SACs, even better than natural enzymes under some reaction conditions. In addition, compared with other antioxidant nanozymes of SACs reported in the literature (Supplementary Table 4), such as single-atom Ir enzyme mimics (Ir NC SAzymes)^58^ and single-atom cobalt nanozymes (Co-SAzymes)^59^, FeN_4_O_2_-SACs exhibit superior catalytic activities. It can be inferred that the multienzyme activity is related to the fact that FeN_4_O_2_-SACs have a structure analogous to those of natural anti-inflammatory enzymes.

### Hyperproliferation and inflammatory infiltration inhibition of HaCaT cells by FeN_4_O_2_-SACs via ROS scavenging

To investigate the impact of FeN_4_O_2_-SACs on HaCaT cells, different concentrations of FeN_4_O_2_-SACs were incubated with the cells. It is found that the cell viability decreases with the increasing FeN_4_O_2_-SACs concentration. The IC_50_ of FeN_4_O_2_-SACs on HaCaT cells is determined to be 87.8 μg/ml (Supplementary Fig. 19). Additionally, the cytotoxicity profile of FeN_4_O_2_-SACs on normal human epidermal keratinocytes (NHEK cells) was also evaluated, and its antiproliferation and anti-inflammation effects can be clearly seen (Supplementary Figs. 20, 21).

**Fig. 5:**
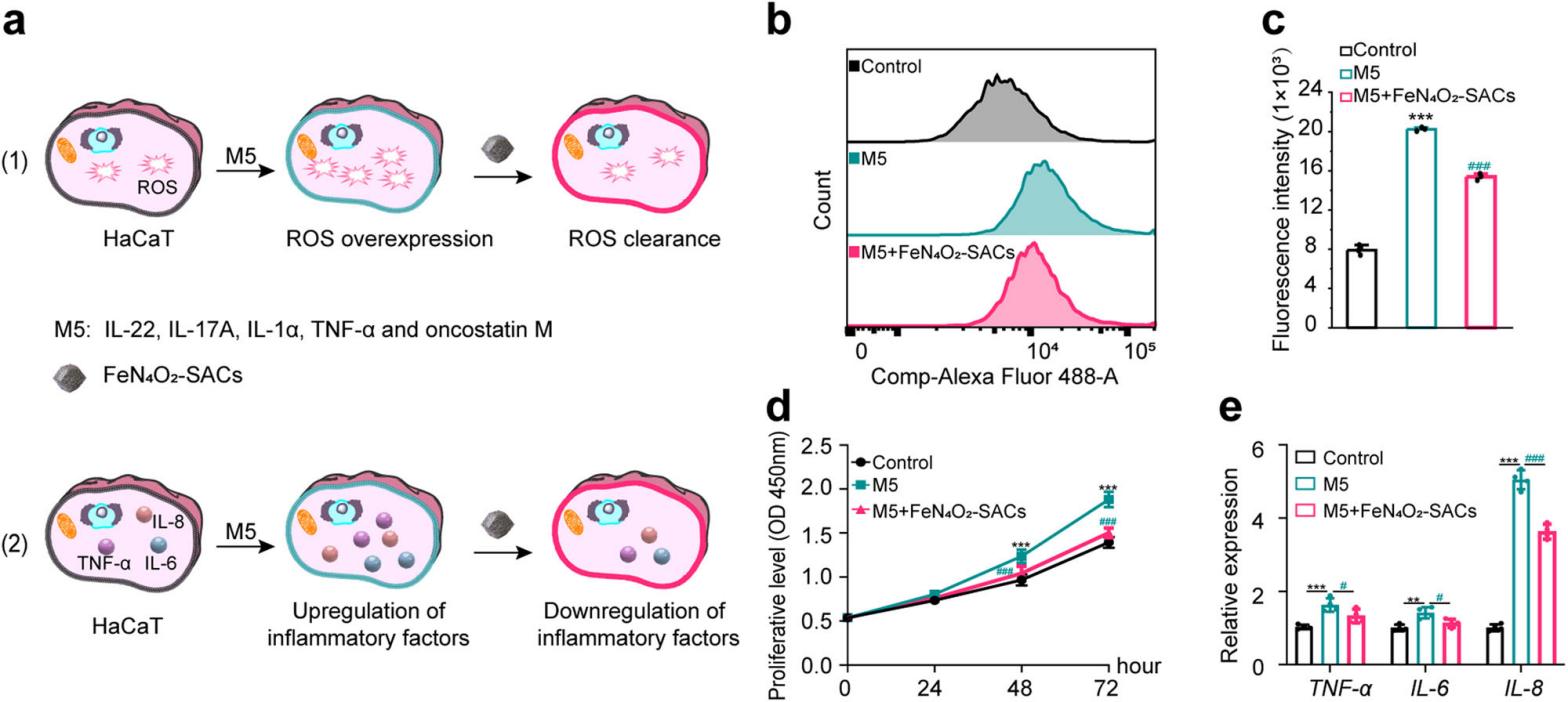
FeN_4_O_2_-SACs effectively inhibit hyperproliferation and inflammation by scavenging ROS in vitro. **a** Schematic illustration of the cell experimental design and results. **b, c** Flow cytometry analysis of HaCaT cells in different groups stained by DCFH-DA (2,7-Dichlorodihydrofluorescein diacetate, EX498/EM530 nm) (**b**) and the quantification of fluorescence intensity (**c**). *n* = 3 independent experiments and data are presented as mean ± SD. **d** CCK-8 assay showing the cell proliferative viabilities of different groups*.* *n* = 3 independent experiments and data are presented as mean ± SD. **e** RT-qPCR results for the inflammatory factors *TNF-α*, *IL-6*, and *IL-8* at 24 hours. *n* = 4 independent experiments and data are presented as mean ± SD. ***p < 0.001, **p < 0.01 versus the Control group; ^###^*p* < 0.001, ^#^*p* < 0.05 versus the M5 group. Statistical significance was calculated via one-way ANOVA (**c**), two-way ANOVA (**d**), and two independent samples unpaired Student’s *t* test (**e**). Source data are provided as a Source Data file.

Since the oxidative damage induced by ROS contributes to the development of psoriasis, the effect of FeN_4_O_2_-SACs on total cellular ROS levels was first detected by flow cytometry (Fig. 5a–c and Supplementary Fig. 22) and fluorescence microscope (Supplementary Fig. 23), which was based on the knowledge that ROS can oxidize nonfluorescent DCFH to generate fluorescent DCF. In untreated HaCaT cells, FeN_4_O_2_-SACs addition can hardly affect the normal ROS level. Interestingly, in contrast to the overactive ROS production induced by M5 (a composition of five proinflammatory factors containing IL-22, IL-17A, IL-1α, TNF-α, and oncostatin M) in HaCaT cells, there is a great decrease in ROS accumulation in the M5 + FeN_4_O_2_-SACs group. Similarly, reductions in ROS are observed in NHEKs by flow cytometry (Supplementary Fig. 24), which is thought to be advantageous to psoriasis therapy.

To further determine whether FeN_4_O_2_-SACs could function in psoriasis treatment or not in vitro, the upregulated proliferation of HaCaT cells and inflammation were first established in M5-treated HaCaT cells, followed by FeN_4_O_2_-SACs treatment. Attractively, the FeN_4_O_2_-SACs treatment has largely weakened the M5-stimulated psoriasis-like morbidity status and shows inhibited cell proliferation and reduced *TNF-α*, *IL-6*, and *IL-8* mRNA expressions (Fig. 5d, e). It is suggest that FeN_4_O_2_-SACs can remarkably rescue the hyperproliferation and excessive inflammation of psoriasis.

### Alleviation of skin lesions in IMQ-induced psoriasis-like dermatitis mice

**Fig. 6:**
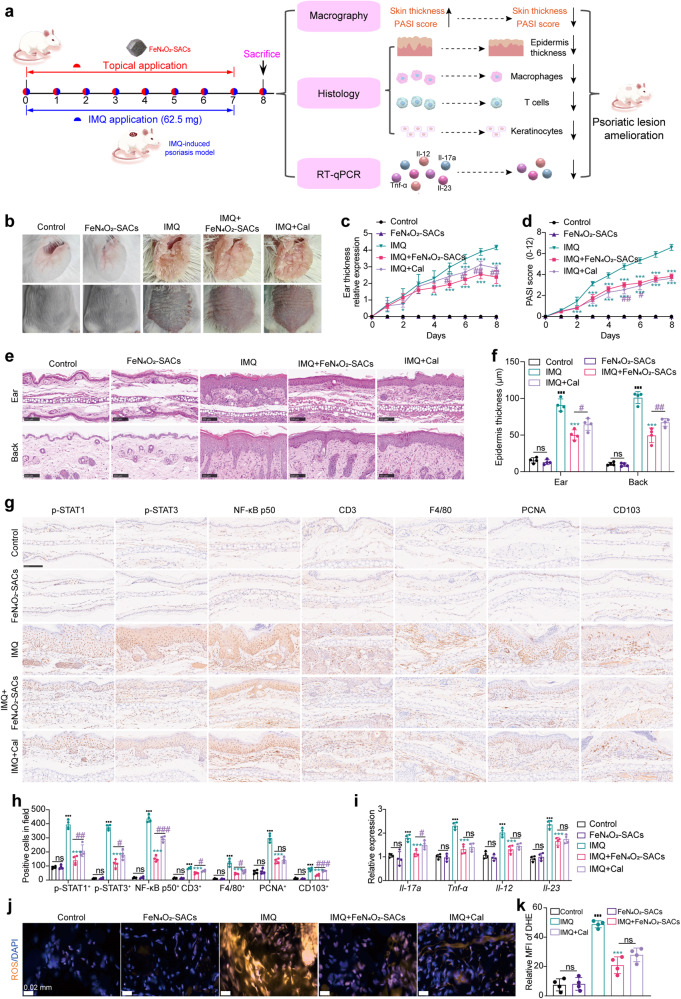
FeN_4_O_2_-SACs effectively alleviate the psoriasis dermatitis in vivo. **a** Schemes of the IMQ-induced psoriasiform model, treatments and efficacy. **b** Representative images of lesions on day 8, *n* = 4 samples/group. **c**, **d** Quantification of ear thickness (**c**) and PASI score (**d**), *n* = 4 samples/group and data are presented as mean ± SD. **e**, **f** Histological staining (**e**) and the corresponding quantifications (**f**). Scale bar=100 μm, *n* = 4 samples/group and data are presented as mean ± SD. **g**, **h** Levels of inflammatory signals, T cells, macrophages, proliferative keratinocytes and tissue-resident memory T cells analyzed by immunohistochemistry labelling of p-STAT1, p-STAT3, NF-κB p50, CD3, F4/80, PCNA and CD103 in the ear lesions on day 8 (**g**), and the corresponding quantifications (**h**). Scale bar=100 μm, n = 4 samples/group and data are presented as mean ± SD. **i** The mRNA expression levels of *Il-17a, Tnf-α, Il-12*, and *Il*-*23* at the ear skin tissues detected by RT-qPCR. *n* = 4 samples/group and data are presented as mean ± SD. **j**, **k** The ROS level of ear skins from different groups detected by fluorescence probe DHE (Dihydroethidium, Filter block of spORANGE at EX532-554/EM576-596 nm) (**j**) and the quantification (**k**) of relative mean fluorescence intensity (MFI), orange color indicates the ROS-positive, n = 4 samples/group and data are presented as mean ± SD. ^***^p < 0.001, ^**^p < 0.01, ^*^p < 0.05 versus the IMQ group; ^###^*p* < 0.001, ^##^*p* < 0.01, ^#^*p* < 0.05 versus the IMQ+Cal group; ^■■■^*p* < 0.001 versus the Control group; ns means no significance. Statistical significance was calculated via two-way ANOVA (**c**, **d**), two independent samples unpaired Student’s *t* test (**f**, **h**, **i**, **k**). Source data are provided as a Source Data file.

To confirm the therapeutic effects of FeN_4_O_2_-SACs on psoriasis lesions, we utilized imiquimod (IMQ) cream to establish psoriasis-like mouse models and then treated them with FeN_4_O_2_-SACs (Fig. 6a). FeN_4_O_2_-SACs at different concentrations (1.25 μg/ml, 2.5 μg/ml, 5 μg/ml) consistently reduce the ear thicknesses, psoriasis area and severity index (PASI) score of mouse lesions, among which 2.5 μg/ml FeN_4_O_2_-SACs showed the most prominent effects and were used in follow-up experiments (Supplementary Fig. 25). As shown in Fig. 6b, the erythematosquamous plaques induced by IMQ have been more effectively alleviated by FeN_4_O_2_-SACs treatment than by calcipotriol (Cal), and the ear thickness and PASI score are also decreased by the FeN_4_O_2_-SACs treatment (Fig. 6c, d). Microcosmically, FeN_4_O_2_-SACs relieve IMQ-induced epidermal hyperplasia, acanthosis, and hyperkeratosis with performance in a comparable manner to that of Cal treatment (Fig. 6e, f). Immunohistochemistry staining (IHC) shows that at the lesional skin, the increased numbers of CD3^+^ T cells, F4/80^+^ macrophages, and PCNA^+^ keratinocytes, as well as p-STAT1^+^, p-STAT3^+^ and NF-κB p50^+^ signals^60^ developed in the IMQ group have been strikingly diminished in the FeN_4_O_2_-SACs-treated and Cal groups, and the FeN_4_O_2_-SACs group exhibits a more significant reduction in p-STAT1^+^, p-STAT3^+^, NF-κB p50^+^ signals, CD3^+^ T cells and F4/80^+^ macrophages than the Cal group (Fig. 6g, h). With FeN_4_O_2_-SACs treatment, the signal of tissue-resident memory T cells (indicated by CD103^+^) activated by IMQ induction is weakened. Consistently, the flow cytometry results shows that FeN_4_O_2_-SACs remarkably reduce the CD3^+^T cells, F4/80^+^CD11b^+^macrophages and CD103^+^CD8^+^T_RM_ cells (Supplementary Figs. 26–28)^61^. Also, the mRNA levels of the proinflammatory cytokines *Il-17a*, *Tnf-α*, *Il-12*, and *Il-23* are markedly elevated in the IMQ group but greatly downregulated by FeN_4_O_2_-SACs treatment, similar to the Cal group (Fig. 6i). The anti-inflammatory factor *Il-10* is downregulated by IMQ induction, and FeN_4_O_2_-SACs significantly elevate its level (Supplementary Fig. 29). The in vivo ROS level evaluation reveals that FeN_4_O_2_-SACs treatment significantly ameliorates the oxidative stress induced by IMQ (Fig. 6j, k). Additionally, the organ toxicity of FeN_4_O_2_-SACs in treating psoriasis-like dermatitis was evaluated and revealed no safety concerns (Supplementary Fig. 30). In conclusion, FeN_4_O_2_-SACs demonstrate a significant therapeutic effect on IMQ-induced psoriasis-like dermatitis mice by attenuating keratinocyte abnormality, excessive inflammation, and oxidative damage, showing great application potential in clinical psoriasis treatment without side effects.

### Transcriptional profiles and GSEA analysis of FeN_4_O_2_-SACs-treated mice

**Fig. 7:**
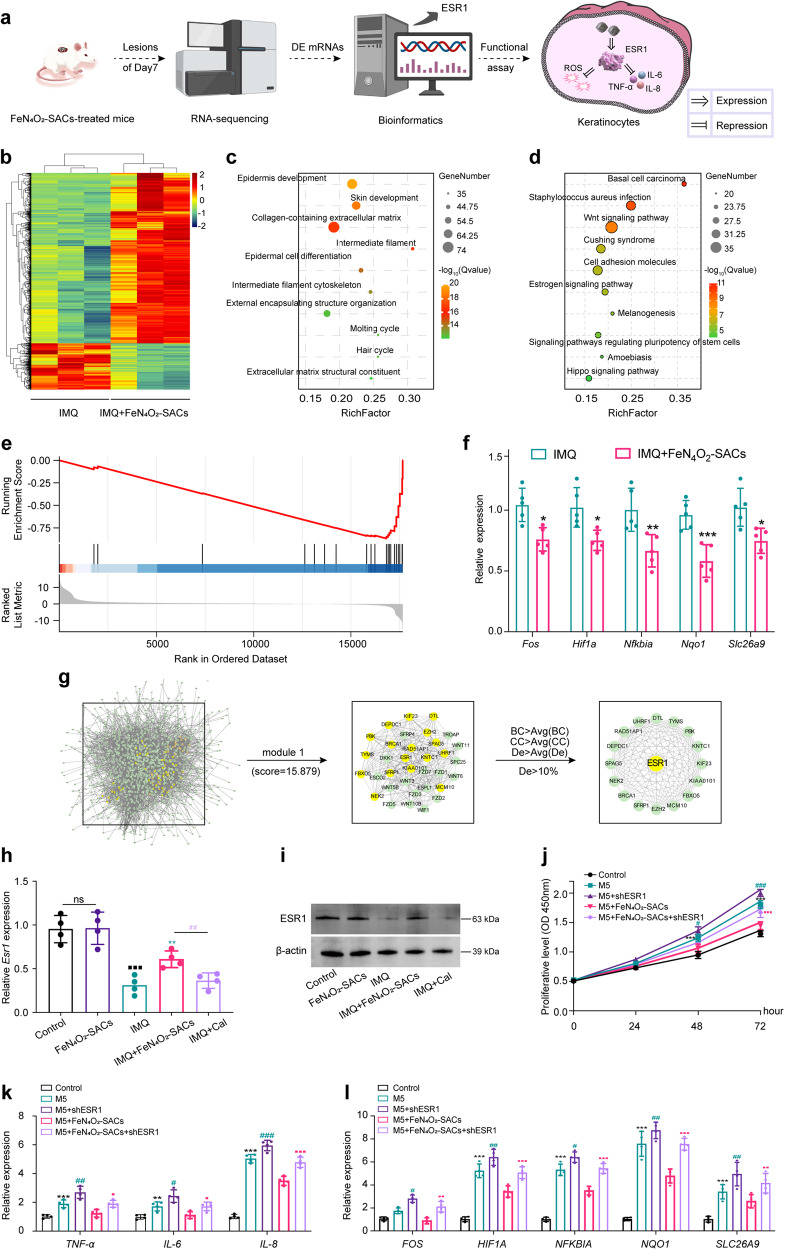
RNA-sequencing of mouse skin lesions and the identification of key protein ESR1. **a** Schematic illustration of the underlying mechanism analysis of FeN_4_O_2_-SACs treatment. **b** The heatmap of differentially expressed (DE) mRNAs (*n* = 3). **c**, **d** GO annotation (**c**) and KEGG pathway enriched (**d**) by DE mRNAs. **e** The enrichment curve of ROS signal (FeN_4_O_2_-SACs versus IMQ). **f** The core genes downregulated in the ear lesions of IMQ+FeN_4_O_2_-SACs group validated by RT-qPCR, *n* = 5 samples/group and data are present as mean ± SD. ^***^p < 0.001, ^**^p < 0.01, ^*^p < 0.05 versus the IMQ group. **g** Protein network of the DE mRNAs. **h** RT-qPCR validation of *Esr1* in ear skin tissues, *n* = 4 samples/group and data are present as mean ± SD. ^**^p < 0.01 versus the IMQ group; ^##^*p* < 0.01 versus the IMQ+FeN_4_O_2_-SACs group; ^■■■^*p* < 0.001 versus the Control group; ns means no significance. **i** Western blotting analysis of ESR1 protein in the ear lesions of mice from different groups. The samples derive from the same experiment and that gels/blots were processed in parallel. **j** CCK-8 assay detecting the cell proliferative activities of different groups. *n* = 3 independent experiments and data are present as mean ± SD. **k** The mRNA expression levels of inflammatory factors *TNF-α*, *IL-6*, and *IL-8* detected by RT-qPCR at 24 hours. *n* = 4 independent experiments and data are present as mean ± SD. **l** Relative mRNA levels of ROS-related genes determined by RT-qPCR at 24 hours. *n* = 4 independent experiments and data are present as mean ± SD. ^***^p < 0.001, ^**^p < 0.01, versus the Control group; ^###^*p* < 0.001, ^##^*p* < 0.01, ^#^*p* < 0.05 versus the M5 group; ^■■■^*p* < 0.001, ^■■^*p* < 0.01, ^■^*p* < 0.05 versus the M5 + FeN_4_O_2_-SACs group^.^ Statistical significance was calculated via two-way ANOVA (j), two independent sam*p*les unpaired Student^’^s t test (**f**, **h**, **k**, **l**). Source data are provided as a Source Data file.

RNA sequencing analyses of lesional skins before and after catalytic therapy were utilized to reveal the regulatory mechanism of FeN_4_O_2_-SACs in treating IMQ-induced psoriasis-like dermatitis (Fig. 7a). As a result, 1546 differentially expressed (DE) mRNAs (*p* < 0.05, |Log_2_Fold-Change | ≥ 2) are identified, involving 1216 upregulated and 330 downregulated mRNAs (Fig. 7b). To assess the functional enrichment of DE mRNAs, GO annotations and KEGG pathways were evaluated. The top 10 GO terms are found to be: epidermal development, skin development, collagen-containing extracellular matrix, intermediate filament, epidermal cell differentiation, intermediate filament cytoskeleton, external encapsulating structure organization, molting cycle, hair cycle, and extracellular matrix structural constituent (Fig. 7c), meanwhile the top 10 KEGG pathways are identified to be: bAsAl cell carcinoma, Staphylococcus aureus infection, Wnt signaling pathway, Cushing syndrome, cell adhesion molecules, estrogen signaling pathway, melanogenesis, signaling pathways regulating pluripotency of stem cells, amoebiasis and Hippo signaling pathway (Fig. 7d). These evidences indicate that FeN_4_O_2_-SACs are able to ameliorate psoriasis-like dermatitis by altering the expressions of various DE mRNAs and functioning in skin/dermal development and inflammatory cascades.

To address the essential role of FeN_4_O_2_-SACs in regulating ROS, Gene Set Enrichment Analysis (GSEA) was performed to acquire the ROS-signal enrichment curve by including all the genes regulated by FeN_4_O_2_-SACs. As shown in Fig. 7e, the gene members with greater contribution to the enrichment score are located on the right side of the peak, and the enrichment curve is clustered on the right side. The enrichment score is negative, indicating that compared with the IMQ group, FeN_4_O_2_-SACs administration downregulate the ROS signal pathway. Additionally, we obtained the ROS-related DE mRNAs from KEGG pathways and analyzed them with a *t* test (Supplementary Fig. 31. It can be found that *Fos*, *Hif1a*, *Nfkbia*, *Nqo1*, and *Slc26a9* are significantly downregulated by FeN_4_O_2_-SACs compared with the IMQ group (Fig. 7f). Overall, FeN_4_O_2_-SACs can be applied as an inhibitor of ROS.

### Identification and functional analysis of the key protein ESR1

In order to identify the hub gene of FeN_4_O_2_-SACs that ameliorates psoriatic dermatitis, the Protein-Protein Interaction (PPI) network and important modules were screened from the PPI network. Cytoscape was used to analyze the PPI networks of the cross-targets obtained from String databases. Through Cytoscape’s MCODE plug-in, two closely linked gene modules with degree are identified (module 1, score = 15.879; module 2 (not shown), score = 11.306). A total of 34 targets and 262 interaction pairs are included in module 1, and 50 targets along with 277 interaction pairs are included in module 2. Based on the fact that most of the targets are found in module 1, we performed a topological network analysis to evaluate the regulatory relationship between targets. Topological parameters were used to screen a 40-node, 774-edge subnetwork: BC > Avg (BC), CC > Avg (CC), and De > Avg (De). A kernel network was extracted based on the subnetwork in accordance with De ranking. It consists of 17 nodes and 105 edges, with ESR1 being the core node (Fig. 7g). The *ESR1* gene encodes an estrogen receptor (ESR) related to growth, sexual development, and metabolism^62^. ESR1 is significantly reduced in lesions of psoriatic patients^63,64^, and psoriatic inflammation exacerbates in mice with neutrophils and macrophages lacking ESR (*Esr1*^f/f^*Esr2*^f/f^*LysM-*Cre^+^ mice)^65^. Herein, we find that the mRNA and protein levels of ESR1 are downregulated in psoriasiform skin tissues of mice, whereas FeN_4_O_2_-SACs rescue the IMQ-induced ESR1 under-expression (Fig. 7h, i).

By silencing ESR1 with shRNA (shESR1, Supplementary Fig. 32), aggravated cell proliferation and inflammation are observed in M5-induced psoriasis-like in vitro model (Fig. 7j, k), and notably, ROS-related hub gene upregulations can be seen (Fig. 7l). Then FeN_4_O_2_-SACs treatment downregulates the relative mRNA levels of ROS-related genes to large extents, which alleviated the inflammation. Further, ESR1 expression reductions (M5 + FeN_4_O_2_-SACs + shESR1) again reverse the amelioration effects over hyperproliferation, excessive inflammation and ROS generation by FeN_4_O_2_-SAC treatment, resulting in keratinocyte hyperproliferation, inflammatory cytokine overexpression and ROS-associated gene upregulations. Conversely, when the expression of ESR1 was knocked down using CRISPR/Cas9, FeN_4_O_2_-SACs treatment is not able to rescue the hyperproliferation and undue inflammation of M5-induced psoriasis-like in vitro model, indicating that ESR1 is indispensable for the curative effects of FeN_4_O_2_-SACs (Supplementary Fig. 33). In summary, FeN_4_O_2_-SACs are highly effective in psoriasis control, hyperproliferation restraint, inflammation inhibition and ROS reduction by upregulating ESR1.

### Effective prevention of psoriasis relapse by FeN_4_O_2_-SACs

**Fig. 8:**
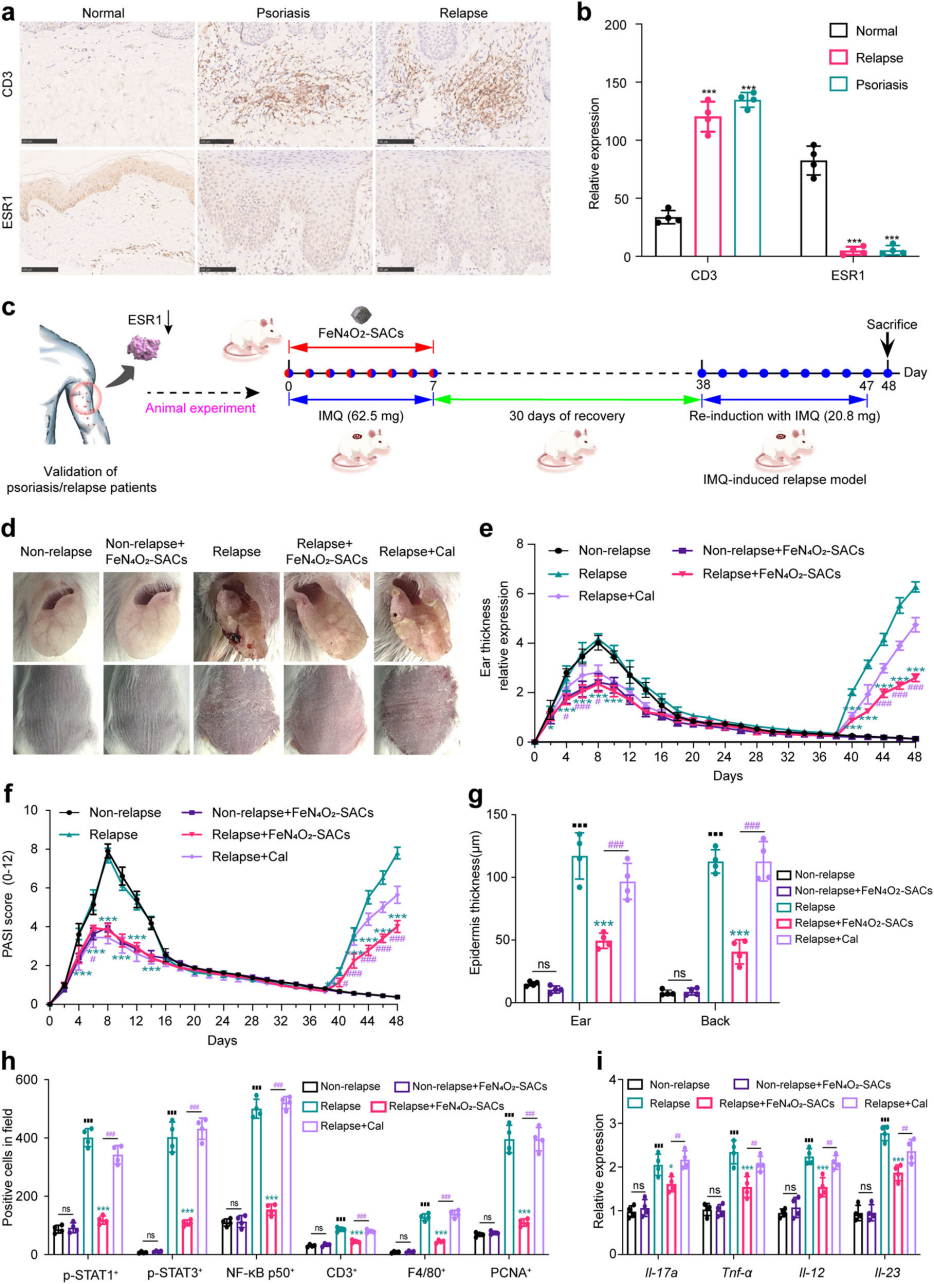
FeN_4_O_2_-SACs suppress the recurrence of psoriasis-like skin dermatitis. **a** IHC stainings of CD3 and ESR1 in skin tissues of normal, psoriatic, and recurrent individuals. Scale bar = 100 μm. **b** Relative quantifications of CD3 and ESR1 determined by ImageJ. *n* = 4 samples/group and data are presented as mean ± SD. ^***^p < 0.001 versus the Normal group. **c** Experimental design of IMQ-stimulated psoriasis relapse model. **d** Representative images of relapse models, *n* = 4 samples/group. **e**, **f** Quantifications of ear thickness (**e**) and PASI score (**f**), *n* = 4 samples/group and data are presented as mean ± SD. **g** Relative quantifications of histological staining. *n* = 4 samples/group and data are presented as mean ± SD. **h**, Quantifications of p-STAT1^+^, p-STAT3^+,^ NF-κB p50^+^ signals, CD3^+^ T cells, F4/80^+^ macrophages, and PCNA^+^ keratinocytes in ear skin lesion tissues from different groups. *n* = 4 samples/group and data are presented as mean ± SD. **i** The mRNA expression levels of *Il-17a*, *Tnf-α*, *Il-12* and *Il-23* of ear skin tissues detected by RT-qPCR. *n* = 4 samples/group and data are presented as mean ± SD. ^***^p < 0.001, ^**^p < 0.01, ^*^p < 0.05 versus the Relapse group; ^###^*p* < 0.001, ^##^*p* < 0.01, ^#^*p* < 0.05 versus the Relapse+Cal group; ^■■■^*p* < 0.001 versus the Non-rela*p*se group; ns means no significance. Statistical significance was calculated via two-way ANOVA (**e**, **f**), two independent samples unpaired Student’s t test (**b**, **g**–**i**). Source data are provided as a Source Data file.

Two datasets (GSE52471, and GSE14905) related to psoriatic patients from the GEO database were adopted to evaluate the clinical relevance of ESR1, and 34 normal samples and 51 psoriasis samples were obtained (Supplementary Fig. 34a, b). By analyzing the expression levels of ESR1 in normal and psoriatic patients, we observe that psoriatic individuals exhibit significant reductions in ESR1 compared with normal individuals (Fig. 8a, b and Supplementary Fig. 34c). Notably, the recurrence of psoriasis is a prevalent and intractable problem in psoriasis therapy. IHC results verify that the ESR1 level is lower in psoriatic and recurrent lesions than in normal skin (Fig. 8a, b), indicating the clinical responsibility of ESR1 deficiency in psoriasis pathology and that FeN_4_O_2_-SACs may ameliorate psoriasis and avoid relapse by restoring ESR1.

We established a relapse model of psoriasis of the mice with secondary but lower doses of IMQ for 10 days after 8-days of treatment and 30-days of rest (Fig. 8c). During the resting period, the dry and rough skin gradually recovered with time in all groups, while after another 10-days of re-stimulation by IMQ, the mice pre-treated with PBS and Cal showed obvious erythematosquamous plaques, and increased epidermal thickness and inflammatory factor levels (*Il-17a*, *Tnf-α*, *Il-12*, *Il-23*). Intriguingly, the skin of previously FeN_4_O_2_-SACs-treated mice exhibits mild psoriasiform lesions and ear thickness compared with the Non-relapse group (the 8-days IMQ-induced psoriasis mice received no secondary IMQ induction with or without FeN_4_O_2_-SACs treatment) (Fig. 8d–g and Supplementary Fig. 35). FeN_4_O_2_-SACs application has largely ameliorated IMQ-induced epidermal thickening and inflammatory infiltration on day 10 after the second stimulation compared with PBS/Cal. IHC results show that in contrast with the increased inflammatory cells and cell proliferation in the Relapse and Relapse+Cal groups, FeN_4_O_2_-SACs treatment significantly diminishes the re-infiltration of p-STAT1^+^, p-STAT3^+^, NF-κB p50^+^ signals, CD3^+^ T cells, F4/80^+^ macrophages, and PCNA^+^ keratinocytes (Fig. 8h and Supplementary Fig. 36). With FeN_4_O_2_-SACs, the inflammatory cytokins *Il-17a*, *Tnf-α*, *Il-12*, *Il-23* are inhibited even with IMQ induction (Fig. 8i). The in vivo ROS detection shows that FeN_4_O_2_-SACs treatment significantly prevents the mice from the damage of oxidative stress (Supplementary Fig. 37). Additionally, the level of ESR1 is sustained by FeN_4_O_2_-SACs compared with the Relapse group (Supplementary Fig. 38). These data suggest that psoriasis catalytic therapy is an effective therapeutic modality for preventing disease rebounding of psoriasis-like skin lesions through ESR1, much better than conventional Cal treatment, in addition to its satisfactory efficacy in the initial treatments.

## Discussion

In this work, a “psoriasis catalytic therapy” strategy has been introduced to ameliorate psoriatic lesions and prevent its recurrence by designing catalytically active biomimetic materials (FeN_4_O_2_-SACs) possessing multiple CAT-, SOD- and APX-like activities to continuously scavenge overexpressed ROS. Notably, the multienzyme-like activities of Fe in FeN_4_O_2_-SACs greatly outperform those of Ce in CeO_2_, Mn in Mn_3_O_4_ and Zn in Zn-SACs by several orders of magnitude (Summary in Supplementary Table 3). As an efficient treatment modality, FeN_4_O_2_-SACs are capable of effectively and continuously inhibiting hyperproliferation and inflammatory infiltration in psoriasis by ROS elimination. Moreover, the PASI score, epidermal thickness, lymphocyte infiltration, macrophage infiltration, proliferation of keratinocytes and levels of proinflammatory cytokines in IMQ-induced psoriasis-like mouse models can be significantly inhibited by FeN_4_O_2_-SACs, demonstrating even better efficacy than that of clinical Cal. Mechanistically, ESR1 deficiency has been identified by RNA sequencing and bioinformatic analysis to play a key role in psoriasis development and relapse, which, attractively, could be inhibited to large extents by FeN_4_O_2_-SACs treatment via ESR1 upregulation. More importantly, a relapse model of psoriasis evidences the significant reductions in psoriasis recurrence by FeN_4_O_2_-SACs treatment. The present catalytic therapy strategy opens up a promising therapeutic pathway for psoriasis amelioration and relapse prevention, which serves as a highly effective example of multienzyme-inspired bionics.

## Methods

### Materials

Zinc nitrate hexahydrate (98%, Zn(NO_3_)_2_·6H_2_O) and ferric nitrate nonahydrate (98%, Fe(NO_3_)_3_·9H_2_O) were purchased from Alfa Aesar. Methanol and ethanol were purchased from Sinopharm. 2-Methylimidazole (99%), superoxide dismutase (≥2500 units/mg protein), and xanthine oxidase (from milk, ≥0.4 units/mg protein) were obtained from Sigma-Aldrich. PBS was obtained from YoBiBio. Catalase (from bovine liver, 20000 units/mg) was obtained from Adamas. Catalase Assay Kit and Total Superoxide Dismutase Kit were purchased from Shanghai Beyotime Biotechnology Co., Ltd. Ascorbic acid, xanthine (98%), hydrogen peroxide (H_2_O_2_, 30 wt%), and sulfuric acid (98%) were purchased from Aladdin Co. Ltd. 5-tert-butoxycarbonyl 5-methyl-1-pyrroline N-oxide (BMPO) were purchased from Shanghai Dojindo Co. Ltd.

### SOD-like activity

The O_2_•^-^ clearance ability of FeN_4_O_2_-SACs and natural SOD were verified by ESR measurements. Typically, 31.25 μg/ml Fe-SAs/NC or 0.49 μg/ml natural SOD was added to a mixture of 25 mM BMPO, 1 mM xanthine, and 0.0125 U/mL xanthine oxidase in different pH PBS solutions (pH 6, 7.4, 8).

The SOD-like activity of FeN_4_O_2_-SACs, NC, CeO_2_, and Mn_3_O_4_ was further quantified using a SOD assay kit (WST-8) according to the manufacturer’s instructions. Specifically, O_2_•^-^ is commonly generated through the action of xanthine and xanthine oxidase, and further reacts with WST-8 to form the formazan dye with a characteristic absorption at 450 nm. Hence, the inhibition rate of O_2_•^-^ is available by colorimetric analysis of the WST-8 product. Correspondingly, various samples with concentrations of 15.625, 31.25, and 62.5 μg/ml were added to an equal volume of detection reagent. After approximately 30 min of incubation, the characteristic absorption change at 450 nm was detected using SpectraMax M2 Molecular Devices. The inhibition percentage was determined based on the following equation: inhibition (%) = [(A_0_-A)/A_0_] × 100%, where A is the absorbance of various samples and A_0_ is the absorbance of the control. The inhibition rate of natural SOD (0.12–3.9 μg/ml) against superoxide anion was assayed using the same method.

### CAT-like activity

The CAT-like activity assays of FeN_4_O_2_-SACs and natural CAT were verified by measuring the decline in absorbance of H_2_O_2_ at 240 nm (39.4 M^-1^ cm^-1^) on a UV-Vis spectrophotometer operating under kinetic mode^66^. Typically, experiments were carried out in PBS buffer solution (pH range from 3 to 11) with a total volume of 2.5 ml containing FeN_4_O_2_-SACs (125 μg/ml) or natural CAT (62.5 μg/ml) and H_2_O_2_ (10 mM), and the absorbance was collected over time. Meanwhile, kinetic analysis was carried out by varying the concentrations of FeN_4_O_2_-SACs (0–125 μg/ml) or natural CAT (0–75 μg/ml) at fixed concentrations of H_2_O_2_ (10 mM) in PBS solution and by varying the concentrations of H_2_O_2_ (0–20 mM) at fixed concentrations of FeN_4_O_2_-SACs (125 μg/ml) or natural CAT (62.5 μg/ml) under identical conditions.

The CAT-like activity of FeN_4_O_2_-SACs, NC, CeO_2_, and Mn_3_O_4_ was further determined by a Catalase Assay Kit, and the OD520 was recorded by SpectraMax M2 Molecular Devices. A digital picture of H_2_O_2_ elimination was also captured.

### APX-like activity

The ascorbate peroxidase (APX)-like activity of FeN_4_O_2_-SACs, NC, CeO_2_, and Mn_3_O_4_ was verified by monitoring the absorption of ascorbate with H_2_O_2_ by UV–Vis spectroscopy at 290 nm (2800 M^-1^ cm^-1^). The spectra were collected in PBS (pH=7.4) with a total volume of 2.5 ml containing 0.2 mM ascorbate, 0.02 mg/ml various samples, and H_2_O_2_ (10 mM).

### Cell culture and treatment

The human immortal keratinocyte line HaCaT (Cell Lines Service, Eppelheim, 300493) was cultured in DMEM (HG) supplemented with 10% FBS, 100 μg/ml streptomycin, and 100 U/ml penicillin at 37 °C with 5% CO_2_. Normal human epidermal keratinocytes (NHEK cells, No. 340593) were acquired from Bena Culture Collection (Henan, China) and cultured with CM8-1 culture medium that contains 90% EMEM and 10% FBS. For treatments, different concentrations of FeN_4_O_2_-SACs in PBS (2.5 μg/ml, 5 μg/ml, 10 μg/ml, 20 μg/ml, 40 μg/ml, 80 μg/ml, 160 μg/ml, 320 μg/ml) were assayed and 2.5 μg/ml was determined for the subsequent experiments in vitro. For in vitro models of psoriasis, a mixture containing IL-22, IL-17A, IL-1α, TNF-α, and oncostatin M (M5 cocktail, each of 10 ng/ml) was utilized^67,68^.

### In vitro ROS detection

ROS assay Kit (50101ES01, Yeason) was utilized to detect the ROS level in HaCaT cells according to the manufacturer’s instruction. In Brief, cells in different groups were treated with Control, M5, and M5 + FeN_4_O_2_-SACs for 48 h, respectively. DCFH-DA was diluted with serum-free medium and DCFH-DA was added to the cell suspension and incubated for 30 min at 37 °C in the dark. The fluorescence images were visualized and recorded using a fluorescence microscope at 498/530 nm (excitation/emission) and ×200 magnification.

In flow cytometry analysis, propidium iodide solution (Biolegend, 421301) was added and incubated for 5 min to dye the dead cells. The ROS levels in normal human epidermal keratinocytes (NHEK cells) were also measured using the same procedure.

### In vivo ROS detection

The in vivo ROS level was analyzed using BBoxiProbe® DHE ROS detection kit (BB-47051). According to the manufacturer’s instructions, the fluorescence to determine the ROS level in cells was detected by fluorescence microscope at the excitation wavelength of 532–544 nm and the emission wavelength of 576–596 nm. The filter block we used was spOrange, and the intensity of orange fluorescence is proportional to the level of ROS.

### Animals and treatment

Male BALB/c mice aged 6–8 w were provided by Shanghai SLAC Laboratory Animal Co., Ltd., (no. 20220004020279, SYXK (Hu) 2018-0040) and housed under the conditions of 12 h dark/light cycle, an ambient temperature of 22–25 °C and the humidity of 30–70%. Imiquimod (IMQ) cream (H20030129, Sichuan Med-Shine Pharmaceutical Co.,Ltd) was used for psoriasis-like mouse model construction and all therapeutic strategies were performed for 8 days. All mice except those in the control group were topically administered 62.5 mg/mouse IMQ cream on the ears and backs once daily, and then different treatments involving PBS, FeN_4_O_2_-SACs or calcipotriol were administered to the same area 6 h later. For determination of FeN_4_O_2_-SACs consistency, mice were randomly divided into four intervention groups (62.5 mg/mouse IMQ cream and treatment 6 h later): Control group (PBS, 80 μl/mouse), 1.25 μg/ml group (1.25 μg/ml of FeN_4_O_2_-SACs, 80 μl/mouse), 2.5 μg/ml group (2.5 μg/ml of FeN_4_O_2_-SACs, 80 μl/mouse), and 5 μg/ml group (5 μg/ml of FeN_4_O_2_-SACs, 80 μl/mouse). For therapeutic efficacy of FeN_4_O_2_-SACs, mice were randomly arranged into five groups: control group (PBS, 80 μl/mouse), FeN_4_O_2_-SACs group (2.5 μg/ml FeN_4_O_2_-SACs, 80 μl/mouse), IMQ group (PBS 6 h after IMQ, 80 μl/mouse), IMQ+FeN_4_O_2_-SACs group (2.5 μg/ml FeN_4_O_2_-SACs 6 h after IMQ), and IMQ+Cal group (1 mg/kg calcipotriol 6 h after IMQ). To evaluate the effectiveness of FeN_4_O_2_-SACs in decreasing recurrence, psoriasis relapse models were constructed. Mice were recovered for 30 days after 8-day treatment as mentioned above and then rechallenged with reduced doses of IMQ (20.8 mg/mouse) at the same lesion location for another 10 consecutive days. During the intervention period, the ear thickness of all mice was measured by vernier caliper, and the PASI score of lesion skins was calculated. Mice were executed by CO_2_ inhalation suffocation at the indicated timepoint, and the lesion tissues were collected for further analysis (histology, RT-qPCR, flow cytometry, western blotting, ROS detection, and RNA-seq). All the animal procedures were approved by Ethics Committee of Yueyang Hospital affiliated to Shanghai University of Traditional Chinese Medicine (no. YYLAC-2021-107-6, YYLAC-2022-160-3).

### RNA sequencing

IMQ-induced psoriasis-like mice treated with or without FeN_4_O_2_-SACs were executed on day 8 and the lesional skin tissues were lysed with TRIzol to isolate the total RNA. mRNA selection, fragmentation, cDNA synthesis, and library preparation were performed for each sample, and the TruSeq stranded mRNA LT sample preparation kit (RS-122-2103, Illumina) was used for sequencing. RNA sequencing analysis was conducted by Shanghai Biochip Co., Ltd.. After DE mRNAs screening (*p* value < 0.05, |Log2Fold-change | ≥ 1), the Gene Ontology (GO) enrichment analysis was carried out through Fisher’s test, and the significant enrichment pathways of these DE mRNAs were determined through the Kyoto Encyclopedia of Genes and Genomes database (KEGG). Additionally, to uncover a more comprehensive biological pathways, the R-package fgsea was employed to conduct the Gene Set Enrichment Analysis (GSEA) on the entire set of genes. This package incorporated both the preranked gene list, obtained from the Molecular Signature Database. The fgsea result was subjected to filtration based on the criterion that the pathway’s adjusted *p*-value was less than 0.05. The visualization of the KEGG and GSEA pathways was conducted using ggplot2. OE Biotech Co., Ltd (Shanghai, China) provided technical supports for bioinformatic analyses. The raw data of RNA-sequencing has been deposited in the Entrez Molecular Sequence Database System under BioProject accession number of PRJNA909970 (https://www.ncbi.nlm.nih.gov/bioproject/PRJNA909970/).

### Clinical sample

For clinical relevance evaluation of ESR1, healthy individuals and psoriasis patients at both incipient and relapse stages were included with the approval of the Ethics Committee of Yueyang Hospital affiliated to Shanghai University of Traditional Chinese Medicine (no. 2019-29). Skin samples were collected and used for immunohistochemistry (IHC) staining. Written informed consent was obtained from all participants.

### Statistical analysis

Filter and standardize the data before statistical analysis. All values in this study are expressed as mean ± SD. We used Students’ *t*-test to analyze the differences between two groups, one-way ANOVA for multiple comparisons and two-way ANOVA for variables repeated at different time points. Statistics were analyzed and displayed by Graphpad Prism 8. The significant differences were determined by ^***^p < 0.001, ^**^p < 0.01, ^*^p < 0.05; ^###^*p* < 0.001, ^##^*p* < 0.01, ^#^*p* < 0.05; ^∎∎∎^*p* < 0.001, ^∎∎^*p* < 0.01, ^∎^*p* < 0.05.

Other experimental methods are detailed in Supplementary Information.

### Names and symbols of genes and proteins

*ESR1*, human gene; *Esr1*, mice gene; ESR1, protein coded by gene^69,70^.

### Reporting summary

Further information on research design is available in the Nature Portfolio Reporting Summary linked to this article.

### Supplementary information

Supplementary Information

Reporting Summary

Peer Review File

### Source data

Source data file

## Data Availability

All the data supporting the findings of this study are available within the article, source data, and its supplementary information files. Source data are provided as a Source Data file. The raw data of RNA-sequencing has been deposited in the Entrez Molecular Sequence Database System under BioProject accession number of PRJNA909970. This study uses publicly available data from the Protein Data Bank (PDB) under accession codes: 1DGF, 1V0H, and 1AVM. Source data are provided with this paper.
