# Supplementary material for: Single-atom catalysts-based catalytic ROS clearance for efficient psoriasis treatment and relapse prevention via restoring ESR1
Source: Nat Commun. 2023 Oct 25;14:6767. doi: 10.1038/s41467-023-42477-y (PMC10600197; doi:10.1038/s41467-023-42477-y)
Supplement: Supplementary file 1 — Supplementary Information [file 41467_2023_42477_MOESM1_ESM.pdf]

Supplementary information

Single-atom Catalysts-based Catalytic ROS Clearance for efficient psoriasis treatment and relapse prevention via restoring ESR1

Xiangyu Lu[1,4,8], Le Kuai[5,6,8], Fang Huang[1,3,8], Jingsi Jiang[2], Jiankun Song[2], Yiqiong Liu[2], Si Chen[1,4], Lijie Mao[1,4], Wei Peng[7], Ying Luo[5,6], Yongyong Li[2], Haiqing Dong[3], Bin Li[2,6*], Jianlin Shi[1,4*]*

[1]Shanghai Tenth People's Hospital, Shanghai Frontiers Science Center of Nanocatalytic Medicine, Clinical Center For Brain And Spinal Cord Research, School of Medicine, Tongji University, Shanghai 200092, China.

[2]Shanghai Skin Disease Hospital, School of Medicine, Tongji University, Shanghai 200443, China.

[3]Key Laboratory of Spine and Spinal Cord Injury Repair and Regeneration, Ministry of Education, Tongji Hospital, School of Medicine, Tongji University, Shanghai 200065, China.

[4]Shanghai Institute of Ceramics, Chinese Academy of Sciences; Research Unit of Nanocatalytic Medicine in Specific Therapy for Serious Disease, Chinese Academy of Medical Sciences, Shanghai 200050, China.

[5]Department of Dermatology, Yueyang Hospital of Integrated Traditional Chinese and Western Medicine, Shanghai University of Traditional Chinese Medicine, Shanghai 200437, China.

[6]Institute of Dermatology, Shanghai Academy of Traditional Chinese Medicine, Shanghai 201203, China.

[7]Institute of Waste Treatment and Reclamation, College of Environment Science and Engineering, Tongji University, Shanghai 200092, China.

[8]These authors contributed equally: Xiangyu Lu, Le Kuai, Fang Huang

E-mail: jlshi@mail.sic.ac.cn, lib@shskin.com, inano_donghq@tongji.edu.cn

The supplementary information includes:

1. Supplementary Methods
2. Supplementary Figures (Fig. 1-38)
3. Supplementary Tables (Table 1-5)
4. Supplementary Discussion
5. Supplementary References

1. Supplementary Methods

Synthesis of Fe-MOF and FeN_4O_2-SACs

Typically, a 100 ml homogeneous methanol solution of $Zn(NO_3)_2·6H_2O$ (2.793 g, 9.39 mmol) and $Fe(NO_3)_3·9H_2O$ (0.119 g, 0.49 mmol) was quickly mixed with another 100 ml homogeneous methanol solution of 2-methylimidazole (3.24 g, 39.46 mmol) under stirring. After stirring for 1 h, the mixture was poured into a hydrothermal reactor, sealed, and heated in an air dry oven at 120℃ for 4 h. Then, the reactor was removed and allowed to cool naturally. Finally, the precipitates (designated as Fe-MOF) were collected, washed two times with ethanol, and dried at 60°C in a vacuum oven. Zn-MOF was synthesized similarly using $Zn(NO_3)_2·6H_2O$ (2.94 g, 9.88 mmol) without the addition of $Fe(NO_3)_3·9H_2O$ in the reaction system.

To obtain iron single-atom catalysts (FeN_4O_2-SACs) and NC, the prepared Fe-MOF and Zn-MOF powders were pyrolyzed at 800°C for 3 h under N_2 atmosphere with a heating rate of 5 ℃/min in a tube furnace. Subsequently, the pyrolyzed powders were collected separately and dispersed homogeneously in 2 M sulfuric acid (40 ml) with stirring at 80°C for 72 h. As a comparison, FeN_4O_2-SACs were obtained by acid washing in 0.5 M sulfuric acid (40 ml) with stirring at 80°C for 24 h. Finally, the corresponding precipitates were collected separately by centrifugation and dried in a vacuum oven.

Measurements

TEM images were performed on a JEM-2100F transmission electron microscope operated at 200 kV. HAADF-STEM, high-resolution TEM (HRTEM), corresponding energy dispersive X-ray spectroscopy (EDX) mapping, and electron energy loss spectroscopy (ELLS) were performed on a Titan Themis 200 transmission electron microscope. SEM images were obtained on a Hitachi Regulus8100 microscope. XRD patterns were performed on a Rigaku D/MAX-2550 V X-ray powder diffractometer (parameters: Cu Kα, λ=1.5405 Å, 40 mA, and 40 kV). XPS data was carried out at ESCAlab250 (Thermal Fischer). The Fe concentrations of the samples were measured by inductively coupled plasma-optical emission spectrometry (ICP-OES) (Agilent 700 Series, Agilent Technologies). FT-IR spectra were determined by a Nicolet 7000-C spectrometer on KBr pellets as the background correction. Raman spectra were obtained from a Labram HR800-LS55(HORIBA, French). ESR spectra were performed on a JEOL-FA200 ESR spectrophotometer. Nitrogen

adsorption-desorption isotherm was obtained on a Micromeritics TriStar 3020 porosimeter at 77 K to analyze the specific surface area and pore size distribution.

The X-ray absorption spectroscopy (XAS) measurements at the Fe K-edge were collected at the XAS Beamline at the Australian Synchrotron (ANSTO) in Melbourne, Australia. The electron beam energy is 3.0 GeV. With the associated beamline optics (Si-coated collimating mirror and Rh-coated focusing mirror), the harmonic content of the incident X-ray beam was negligible. The beam size was approximately 1×1mm. All spectra were collected under ambient conditions.

^{57}Fe Mössbauer spectrum was measured at a ^{57}Co (Rh) source with activity of 25 mCi. The measurement was carried out using a conventional spectrometer (Germany, Wissel MS-500) at room temperature in transmission geometry with constant acceleration mode. The velocity calibration was performed with a room temperature α-Fe absorber. The spectra were fitted by the software Recoil using Lorentzian Multiplet Analysis.

Transfection

To knock down the expression of ESR1 (RefSeq NM_000125) in HaCaT cells, cells were cultured in puromycin (1 μg/ml) and co-transfected with shRNA-control vectors or shRNA-ESR1 vectors in accordance with the manufacturer's guidelines. The shRNA-ESR1 sequences were obtained from Sigma (https://www.sigmaaldrich.cn/CN/zh/semi-configurators/shrna?activeLink=selectClones): pLKO.1puro-shhESR1-1F,
CCGGGCCCTACTACCTGGAGAACGACTCGAGTCGTTCTCCAGGTAGTAGGGCTT TTTG;
pLKO.1puro-shhESR1-1R,
AATTCAAAAAGCCCTACTACCTGGAGAACGACTCGAGTCGTTCTCCAGGTAGTAGGGC;
pLKO.1puro-shhESR1-2F,
CCGGCTACAGGCCAAATTCAGATAACTCGAGTTATCTGAATTTGGCCTGTAGTTTTTG;
pLKO.1puro-shhESR1-2R,
AATTCAAAAACTACAGGCCAAATTCAGATAACTCGAGTTATCTGAATTTGGCCTGTAG;
pLKO.1puro-shhESR1-3F,
CCGGAGCACCCTGAAGTCTCTGGAACTCGAGTTCCAGAGACTTCAGGGTGCTTT TTTG;
pLKO.1puro-shhESR1-3R,
AATTCAAAAAAGCACCCTGAAGTCTCTGGAACTCGAGTTCCAGAGACTTCAGGGTGCT.

The sequences were cloned into the lentivirus vector pLKO.1 puro with shRNA construct. The enzyme digestion site for directed cloning was 5’-AgeI/EcoRI-3’. The knock-down efficacy of shhESR1-1 was the highest, therefore shhESR1-1 was chosen for the following experiments.

Generation of CRISPR/Cas9-engineered ESR1 knockout HaCaT cells

To knockout (KO) ESR1 in HaCaT cells, single-guide RNA (sgRNA) sequences targeting ESR1were designed using crispor (http://crispor.tefor.net/): KO1, 5’-GTGTGCAATGACTATGCTTC-3’ and KO2, 5’-GGACCATATCCACCGAGTCC-3’. Briefly, HaCaT cells were transfected with the all-in-one plasmid, and the deletion of ESR1 was confirmed according to the manufacturer’s instructions. The KO efficiency was validated through sequencing and western blotting.

CCK-8 assay

The viability of HaCaT cells in different groups was tested by CCK-8 assay[1]. The CCK-8 solution was added at indicated time points and the absorbance was detected at 450 nm. The proliferative level was determined by the absorbance values normalized to the control. Data from three separate experiments are presented as mean ± SD.

EdU assay

BeyoClick^{TM} EdU cell proliferation detection kit (TMB method) was used, and experiment was performed according to the manufacturer’s instruction. Briefly, cells in a 96 well plate were cultured overnight to normal state and then, different treatments were applied (M5 or M5+FeN_4O_2-SACs) for 72 hours. TMB color solution (0.1 ml) was added and incubated at room temperature for 5-30 minutes. The absorbance was measured at 370 nm.

Quantitative Real-Time PCR (RT-qPCR)

The total mRNAs were extracted from the lesional skin biopsies and HaCaT cells using standard TRIzol protocols. RNA concentration and purity were determined by an ultraviolet spectrophotometer at 260/280 absorbance ratios, and the data were analyzed using $2^{-\Delta\Delta CT}$ method. The primers are shown in Supplementary Table 5.

Histology

The skin samples were fixed in 4% formalin for 48 h followed by embedding in paraffin and then stained with hematoxylin and eosin.

For IHC staining, partial sections were stained with CD3 (mice: diluted at 1:200, ab16669, Abcam; human: diluted at 1:100, ab16669, Abcam), F4/80 (mice: diluted at 1:200, #70076, Cell Signaling), PCNA (mice: diluted at 1:1000, NA03-200UG, Sigma), p-STAT1 (mice: diluted at 1:4800, ab109461, Abcam), p-STAT3 (mice: diluted at 1:200, ab76315, Abcam) NF-κB p50 (mice: diluted at 1:18000, ab32360, Abcam), CD103 (mice: diluted at 1:200, ab224202, Abcam) and ESR1 (human: diluted in 1:50, ab32063, Abcam) antibodies. Images were taken under a digital slice scanner at magnification of 20×, and the average number of positive cells in each group was calculated.

Flow cytometry

The isolation of cells from the skin tissues of mice was made following the previous method[2]. In flow cytometry, cells were stained with fluorescently labeled antibodies directly after cell processing. For experiments with murine tissues, the following mAbs were used for extracellular staining: CD45-BV421(30-F11, Biolegend, 103134), CD3-FITC (17A2, Biolegend, 100204), CD4-PerCP (RM405, Biolegend, 100538), CD8-PE (53-6.7, Biolegend, 100708), F4/80-APC (BM8, Invitrogen, 17-4801-82), CD11b-BV785 (M1/70, Biolegend, 101243), Viability-APC-Cy7 (Invitrogen, 65-0865-14), and CD103-BV510 (2E7, Biolegend, 121423). Samples were acquired on the BD LSR Fortessa X-20 equipped with four lasers (BD Biosciences) and data were analyzed using FlowJo software (Ashland, OR).

Bioinformatic analysis

For data acquisition, two psoriasis-related datasets (GSE52471, https://www.ncbi.nlm.nih.gov/geo/query/acc.cgi?acc=GSE52471 and GSE14905, https://www.ncbi.nlm.nih.gov/geo/query/acc.cgi?acc=GSE14905) that involve 34 normal samples and 51 disease samples were obtained from the GEO database (https://www.ncbi.nlm.nih.gov/geo/). The median value was taken as the expression value of genes with multiple probes. The k-nearest neighbor algorithm (R package DMwR2) was used to supplement the missing value, and then the combat function from the R package sva was utilized to adjust the batch effects of two sets.

To screen the internal associations of target sets, we intersected the targets of IMQ+FeN_4O_2-SACs and IMQ groups into Cytoscape 3.7.2. to construct a Protein-Protein Interaction (PPI) network using the Search Tool for the Retrieval of Interacting Genes (STRING) databases (https://string-db.org/) with the highest confidence (0.700). Then, the modules in PPI networks were identified by MCODE algorithm[3] and the parameters of the MCODE were as follows: the degree of cut-off, 2; cluster finding, haircut; node score cut-off, 0.2; k-core, 2; and the maximum depth was 100. Moreover, the CytoNCA plug-in[4] was used to determine the network topology parameters, including Betweenness Centrality (BC), Closeness Centrality (CC), and Degree (De), of the PPI modules.

Western blotting

Protein concentrations were measured by a BCA protein assay kit as referred to the manufacturer's instructions using anti-ESR1 (PA5-16440, Invitrogen).

Enzyme-linked immunosorbent assay (ELISA)

The security indexes of FeN_4O_2-SACs were tested using ELISA kits (Corning). Blood samples were obtained from the mice on the last day of induction.

2. Supplementary Figures

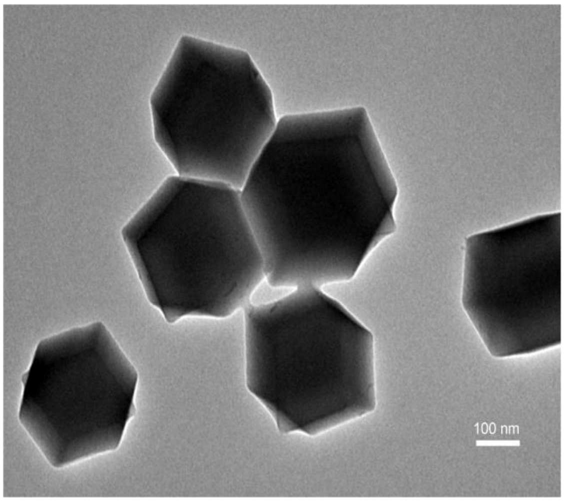

Supplementary Fig. 1. TEM image of Fe-MOF. n = 3 samples with similar results.

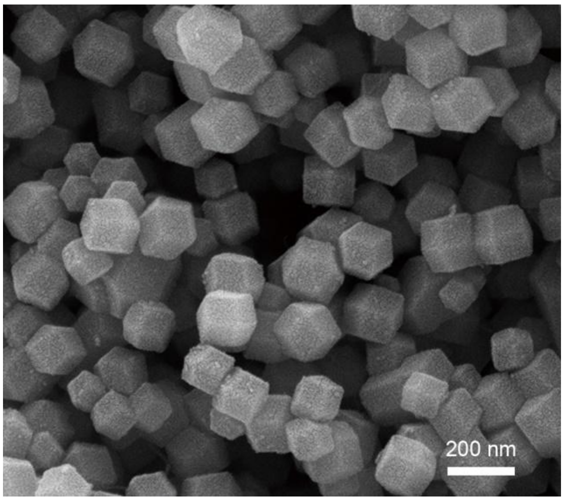

Supplementary Fig. 2. SEM image of FeN_4O_2-SACs. n = 3 samples with similar results.

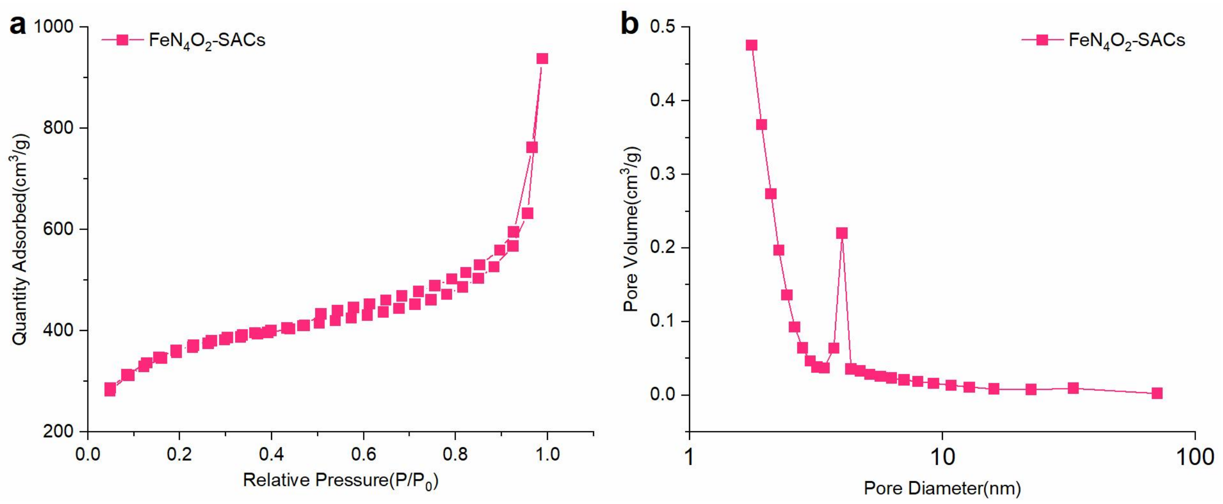

Supplementary Fig. 3. a,**b**, N_2 adsorption-desorption isotherms (**a**) and pore size distribution (**b**) of FeN_4O_2-SACs. Source data are provided as a Source Data file.

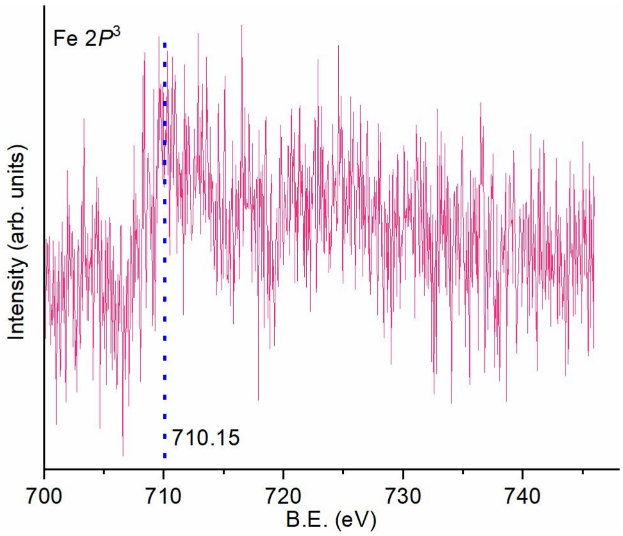

Supplementary Fig. 4. Fe 2p^3 XPS spectrum of the FeN_4O_2-SACs nanoparticle. Source data are provided as a Source Data file.

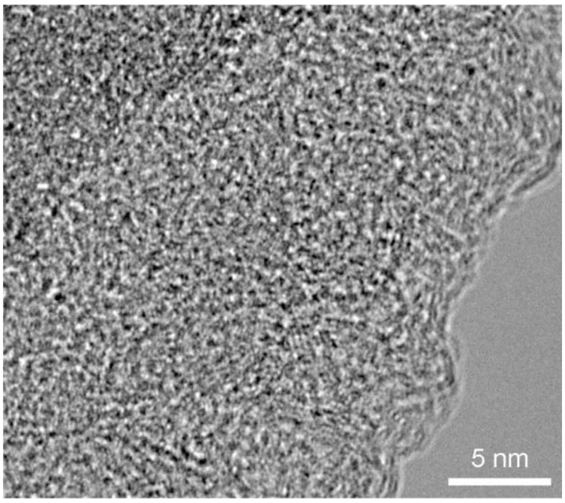

Supplementary Fig. 5. HRTEM image of FeN_4O_2-SACs. n = 3 samples with similar results.

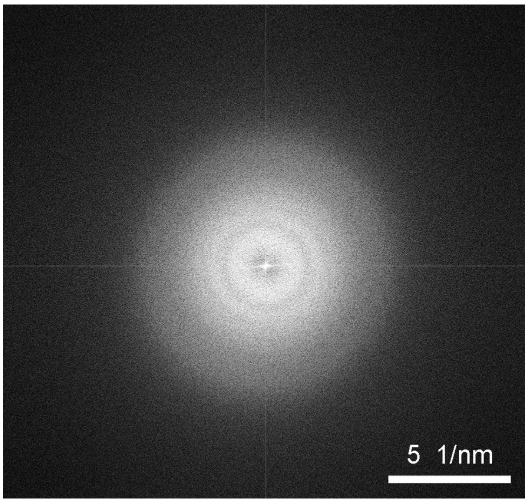

Supplementary Fig. 6. SAED image of FeN_4O_2-SACs. n = 3 samples with similar results.

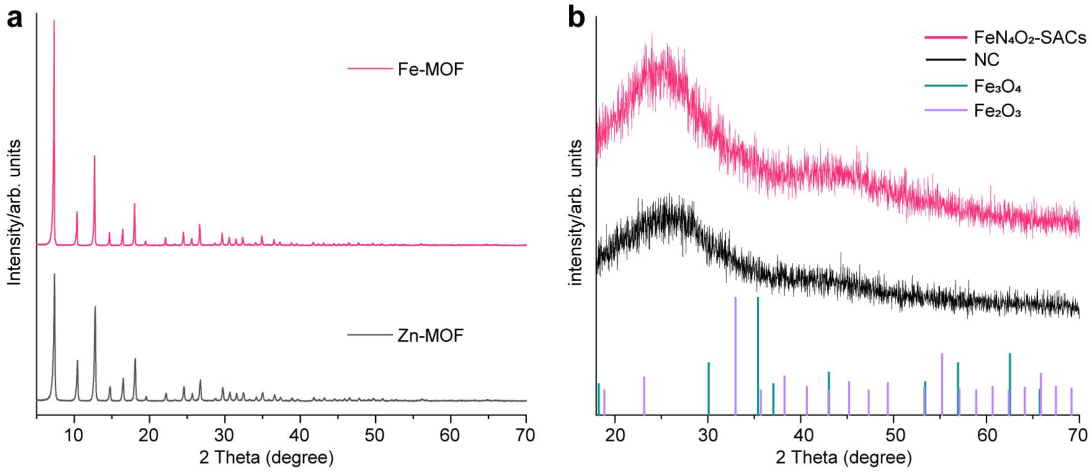

Supplementary Fig. 7. a, XRD patterns of Fe-MOF and Zn-MOF. **b**, XRD patterns of FeN_4O_2-SACs, NC, Fe_2O_3 and Fe_3O_4. Source data are provided as a Source Data file.

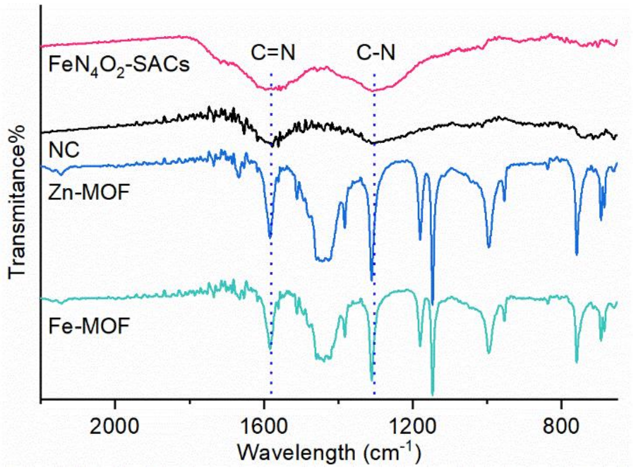

Supplementary Fig. 8. FTIR Spectra of FeN_4O_2-SACs, NC, Zn-MOF, and Fe-MOF. Source data are provided as a Source Data file.

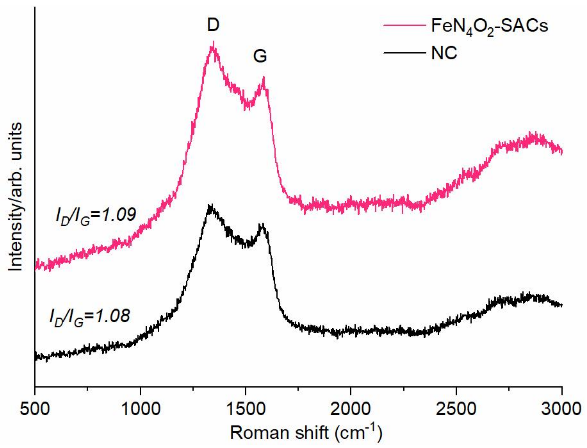

Supplementary Fig. 9. Raman spectra of FeN_4O_2-SACs and NC. Source data are provided as a Source Data file.

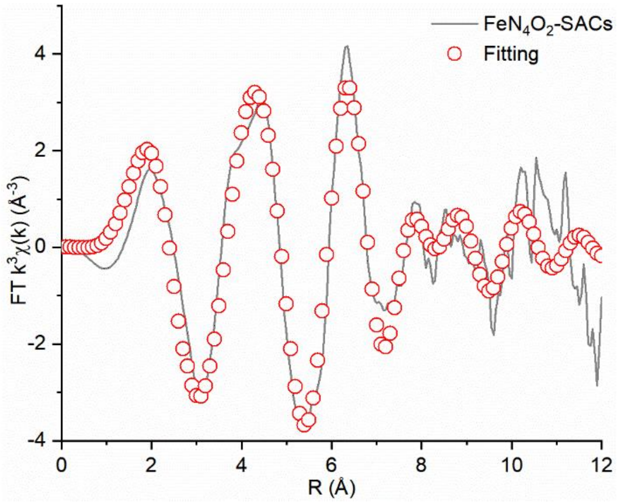

Supplementary Fig. 10. EXAFS fitting in *k* space. Source data are provided as a Source Data file.

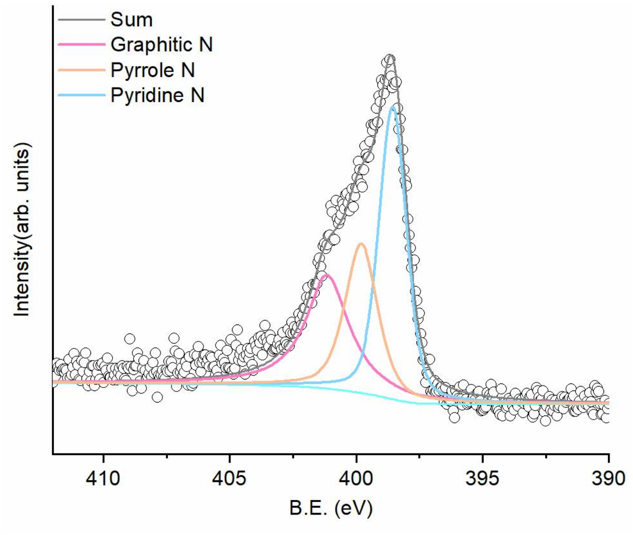

Supplementary Fig. 11. N 1s XPS spectrum of FeN_4O_2-SACs. Source data are provided as a Source Data file.

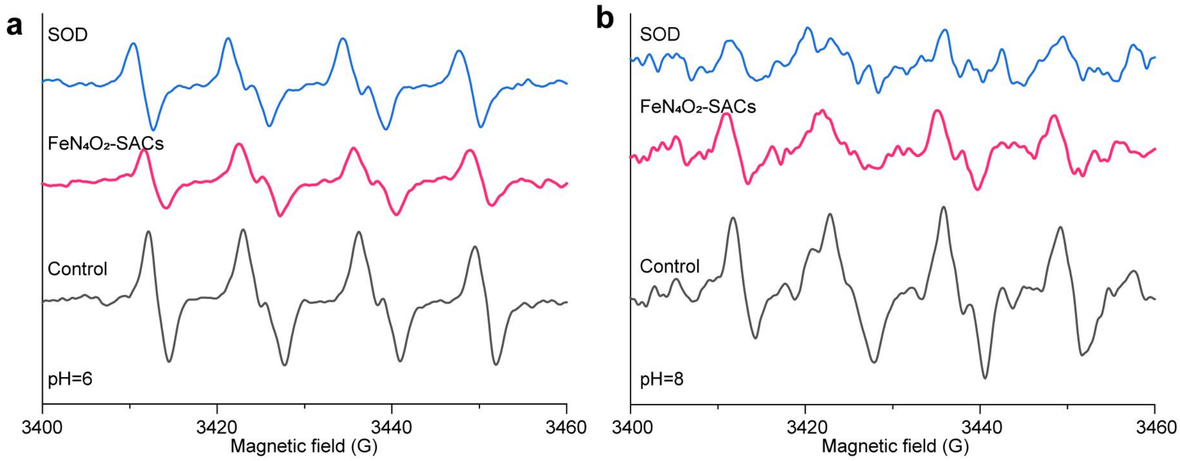

Supplementary Fig. 12. a,**b**, ESR spectra of O_2•⁻ with or without FeN_4O_2-SACs addition and natural SOD at pH=6 (**a**) and pH=8 (**b**). Source data are provided as a Source Data file.

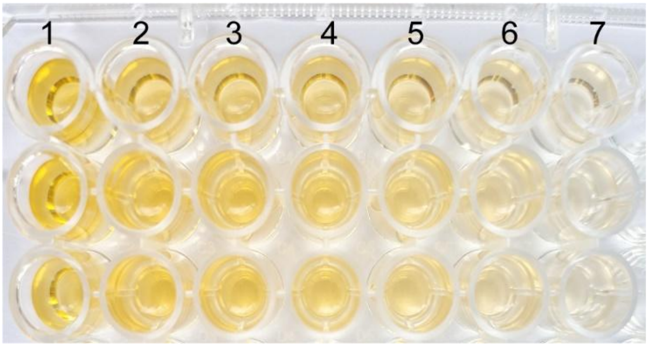

Supplementary Fig. 13. Photographic image illustrating the $O_2•^-$ scavenging capability at varied SOD concentration. 1-7: 0, 0.12, 0.24, 0.49, 0.98, 1.95, 3.91 μg/ml.

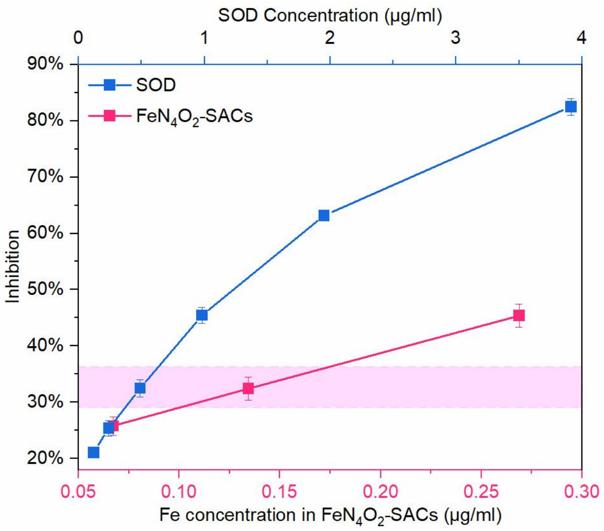

Supplementary Fig. 14. Comparison of the $O_2•^-$ elimination capacity between FeN_4O_2-SACs and natural SOD. n = 3 independent experiments and data are presented as mean ± SD. Source data are provided as a Source Data file.

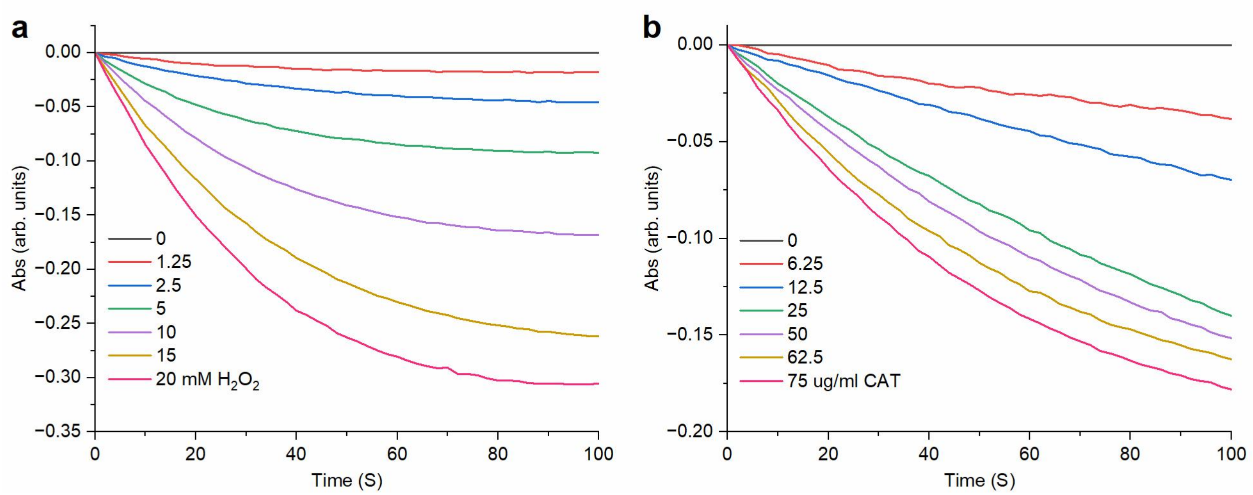

Supplementary Fig. 15. Natural CAT performances. **a**, H_2O_2: 0–20 mM, CAT: 62.5 μg/ml. **b**, CAT: 0–75 μg/ml, H_2O_2: 10 mM. Source data are provided as a Source Data file.

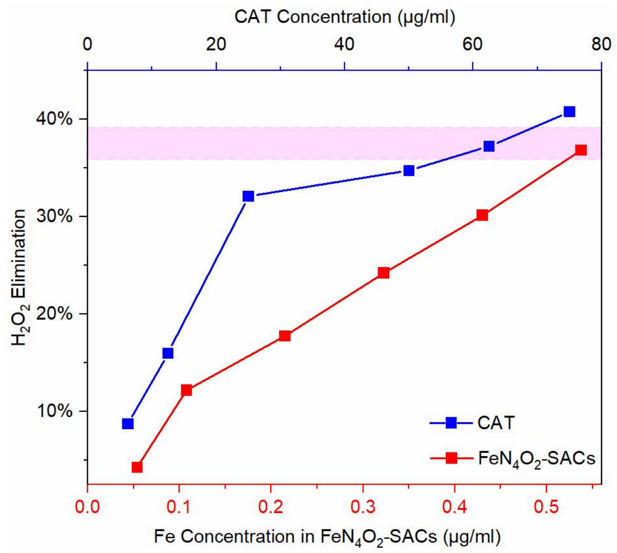

Supplementary Fig. 16. Comparison of H_2O_2 elimination competence between natural CAT and FeN_4O_2-SACs. Source data are provided as a Source Data file.

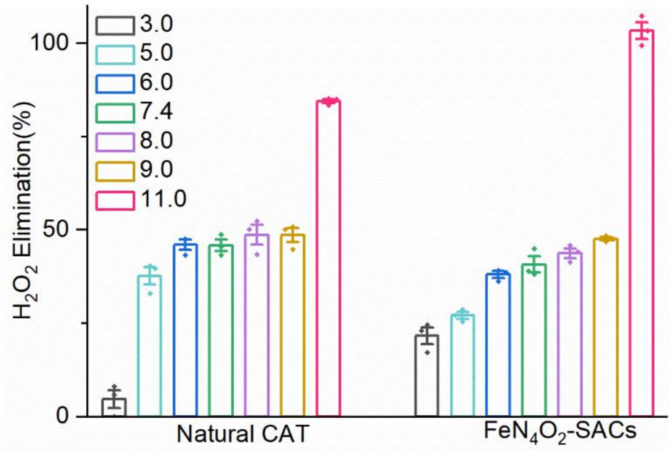

Supplementary Fig. 17. Comparison of H_2O_2 elimination competence between natural CAT and FeN_4O_2-SACs at varied pH values (H_2O_2=10 mM, CAT=62.5 μg/ml, FeN_4O_2-SACs=125 μg/ml). n = 3 independent experiments and data are presented as mean ± SD. Source data are provided as a Source Data file.

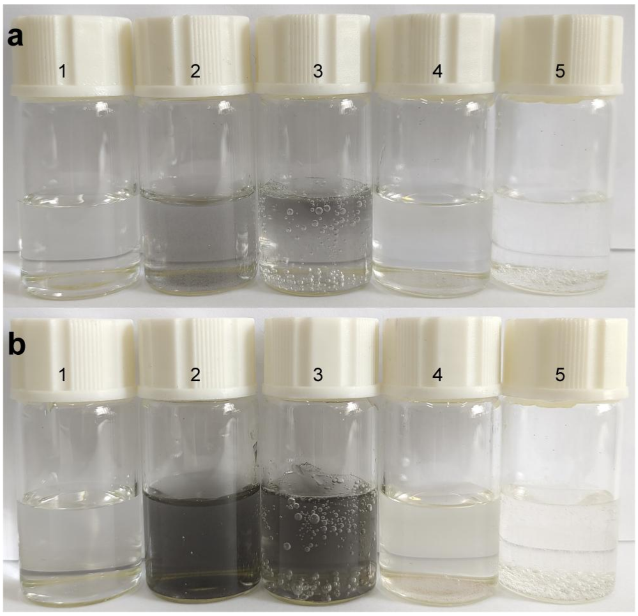

Supplementary Fig. 18. Comparison of the catalytic oxygen production capacity among different samples in the presence of H_2O_2 (1 M). 1: PBS (Control); 2:NC; 3: FeN_4O_2-SACs; 4: CeO_2; 5: Mn_3O_4. **a**, 27 μg/ml; **b**, 53 μg/ml .

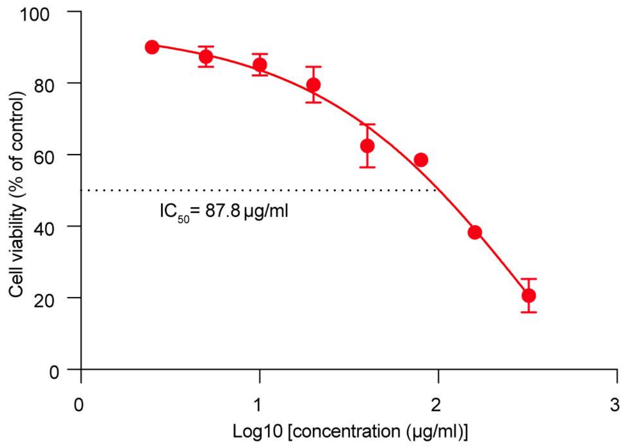

Supplementary Fig. 19. Cytotoxicity of FeN_4O_2-SACs against HaCaT cells in 48 h of incubation. n = 3 independent experiments and data are presented as mean ± SD. Source data are provided as a Source Data file.

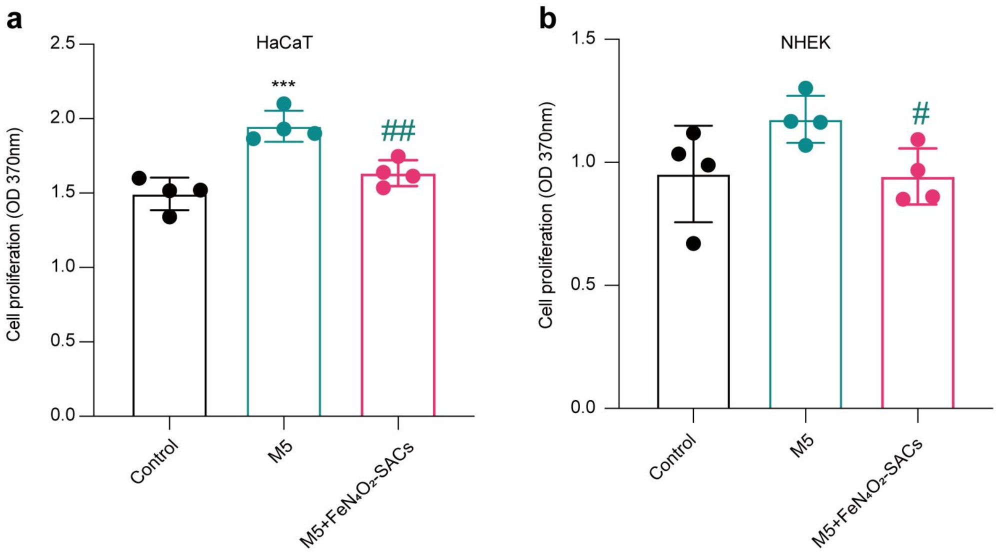

Supplementary Fig. 20. FeN_4O_2-SACs significantly inhibit the cell proliferation of HaCaT cells (**a**) and NHEK cells (**b**). n = 4 independent experiments and data are presented as mean ± SD. ***p<0.001 versus the Control group; ##p<0.01, #p<0.05 versus the M5 group. Statistical significance was calculated via one-way ANOVA. Source data are provided as a Source Data file.

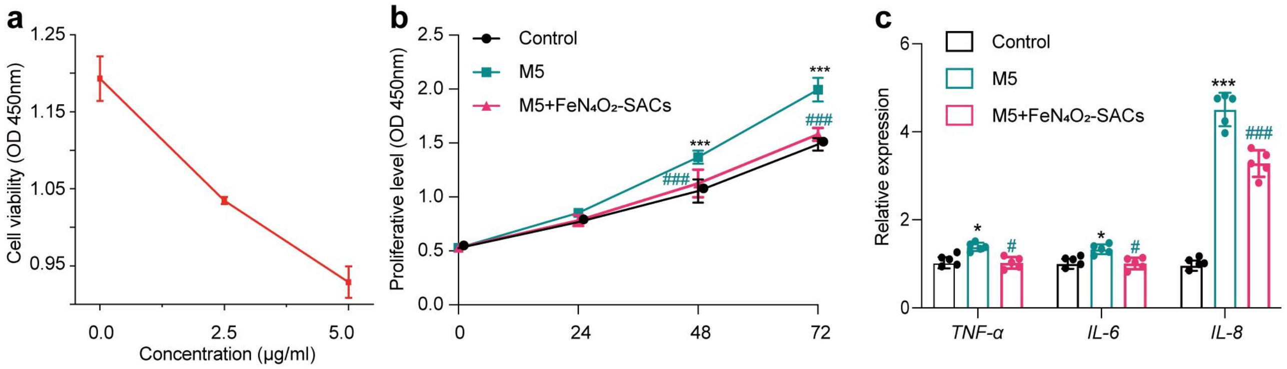

Supplementary Fig. 21. FeN_4O_2-SACs effectively inhibit the hyperproliferation and inflammation of NHEK cells. a, The cytotoxicity profile of FeN_4O_2-SACs on NHEK cells (48 h). n = 3 independent experiments and data are presented as mean ± SD. **b**, CCK-8 assay showing the cell proliferative viabilities of different groups. n = 3 independent experiments and data are presented as mean ± SD. **c**, RT-qPCR results for the inflammatory factors *TNF-α*, *IL-6*, and *IL-8* at 24 hours. n = 5 independent experiments and data are presented as mean ± SD. $^{***}p<0.001$, $^{*}p<0.05$ versus the Control group; $^{\#\#\#}p<0.001$, $^{\#}p<0.05$ versus the M5 group. Statistical significance was calculated via two-way ANOVA (**b**), one-way ANOVA (**c**). Source data are provided as a Source Data file.

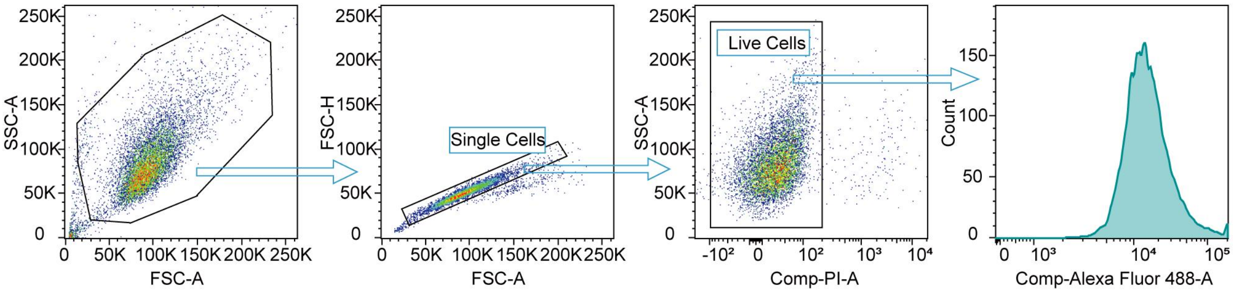

Supplementary Fig. 22. Gating strategy (presented using the M5 group) to determine the intracellular ROS level presented in Fig. 5c.

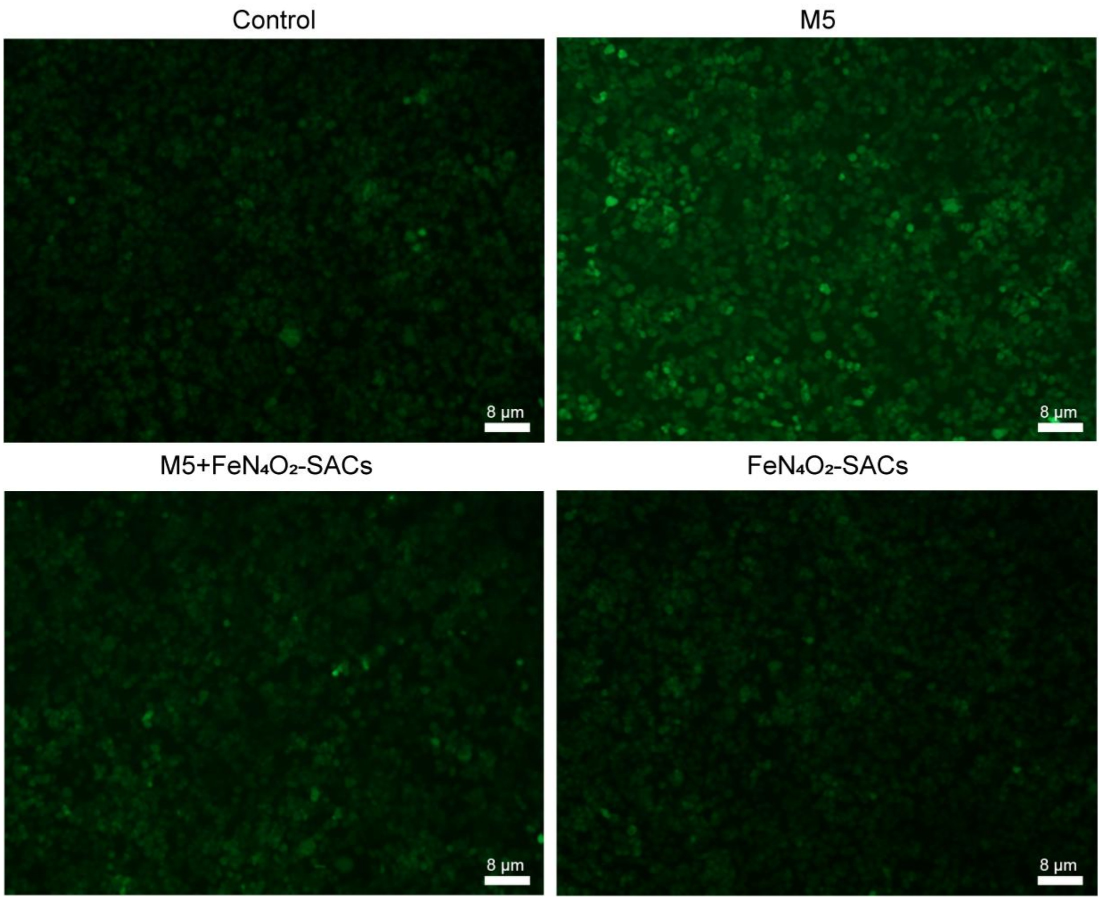

Supplementary Fig. 23. DCFH-DA staining of HaCaT cells in different groups. Representative images captured at ×200 magnification are shown. n = 3 samples/group.

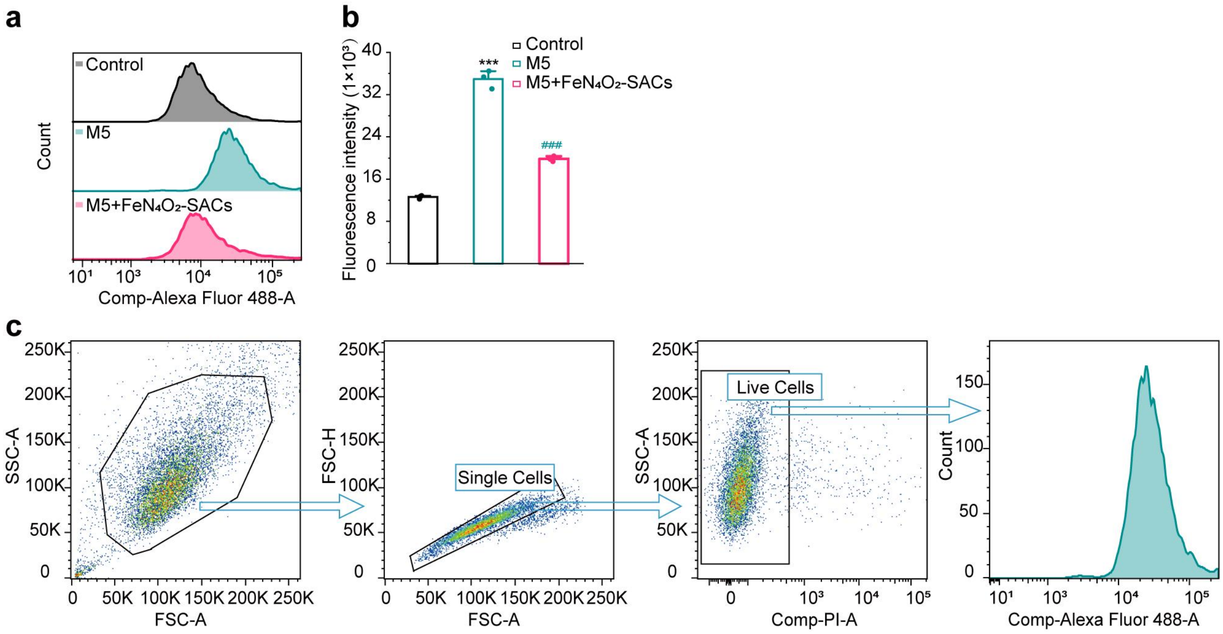

Supplementary Fig. 24. Analysis of ROS levels in NHEK cells. a,**b**, Flow cytometry analysis of NHEKs cells in different groups stained by DCFH-DA (2,7-Dichlorodihydrofluorescein diacetate, EX498/EM530 nm) (**a**) and the quantification (**b**) of fluorescence intensity. n = 3 independent experiments and data are presented as mean ± SD. $^{***}p<0.001$ versus the Control group; $^{\#\#\#}p<0.001$ versus the M5 group. Statistical significance was calculated via one-way ANOVA. **c**, Gating strategy (presented using the M5 group) to determine the intracellular ROS level presented in Supplementary Fig. 24b (presented using the Control group). Source data are provided as a Source Data file.

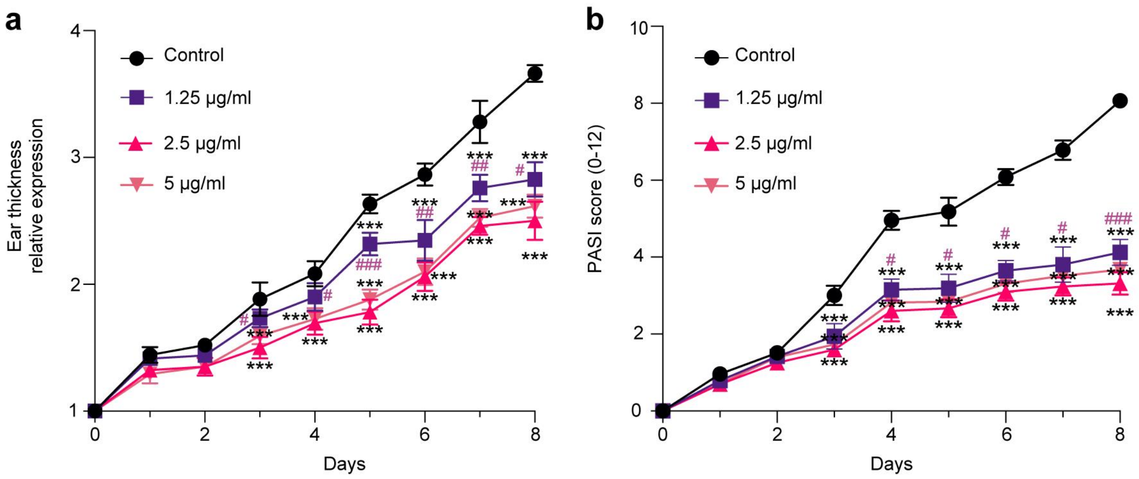

Supplementary Fig. 25. a,**b**, The ear thickness (**a**) and PASI score (**b**) changes of mice at different concentrations of FeN_4O_2-SACs, n = 3 samples/group and data are presented as mean ± SD. $^{***}p$<0.001 versus the Control group; $^{###}p$<0.001, $^{##}p$<0.01, $^{#}p$<0.05 versus the 2.5 μg/ml group. Statistical significance was calculated via two-way ANOVA. Source data are provided as a Source Data file.

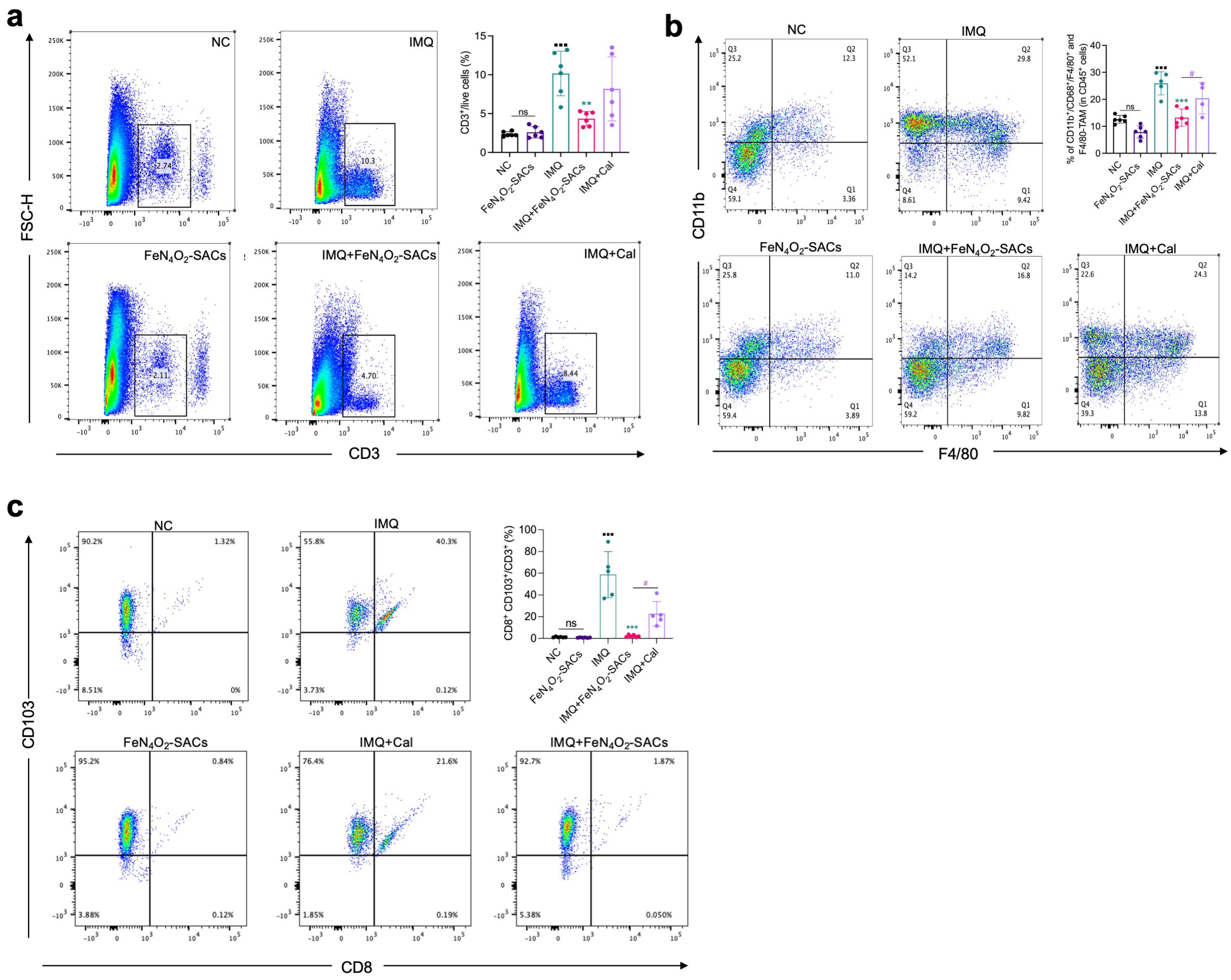

Supplementary Fig. 26. FeN_4O_2-SACs remarkably reduce the numbers of $CD3^+$ T lymphocytes, $CD11b^+F4/80^+$ macrophages and $CD103^+CD8^+T_{RM}$ cells at the ear skins. a, The percentage of $CD3^+$ T lymphocytes in different groups determined by flow cytometry. n = 6 samples/group and data are presented as mean ± SD. **b**, The percentage of $CD11b^+F4/80^+$ macrophages (in $CD45^+$ cells) in different groups quantified by flow cytometry. n = 6 samples/group and data are presented as mean ± SD. **c**, The percentage of $CD103^+CD8^+T_{RM}$ cells (in $CD3^+$ T lymphocytes) in different groups quantified by flow cytometry. n = 5-6 samples/group and data are presented as mean ± SD. $^{***}p<0.001$, $^{**}p<0.01$ versus the IMQ group; $^{\#}p<0.05$ versus the IMQ+Cal group; ■■■$p<0.001$ versus the NC group; ns means no significance. Statistical significance was calculated via one-way ANOVA. Source data are provided as a Source Data file.

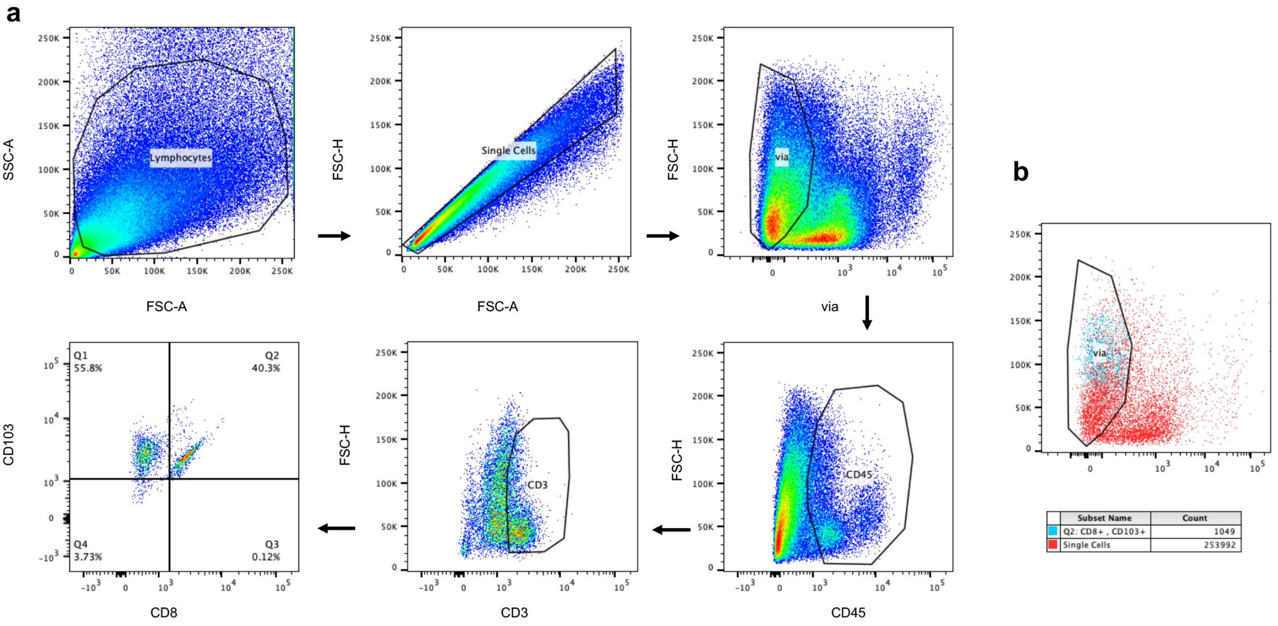

Supplementary Fig. 27. a, Gating strategy applied to CD8$^+$CD103$^+$ T$_{RM}$ cells in Supplementary Fig. 26c. **b**, Flow cytometry plots of live CD8$^+$CD103$^+$ T$_{RM}$ cells among live cells.

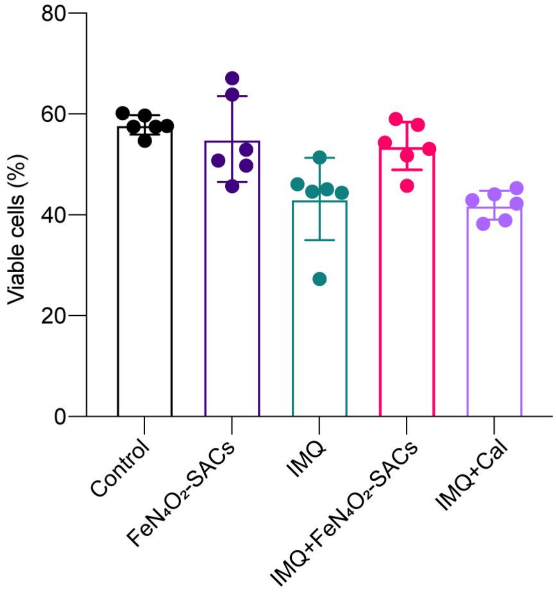

Supplementary Fig. 28. The comparison of cells viability among different groups quantified by flow cytometry. n = 6 samples/group and data are presented as mean ± SD. Statistical significance was calculated via one-way ANOVA. Source data are provided as a Source Data file.

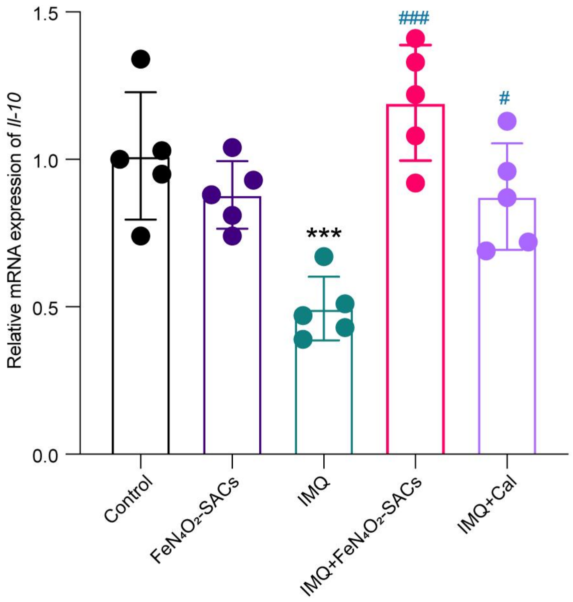

Supplementary Fig. 29. The mRNA expression of *Il-10* in the ear skin lesions of different groups, n = 5 samples/group and data are presented as mean ± SD. $^{***}p<0.001$ versus the Control group; $^{###}p<0.001$, $^{\#}p<0.05$ versus the IMQ group. Statistical significance was calculated via one-way ANOVA. Source data are provided as a Source Data file.

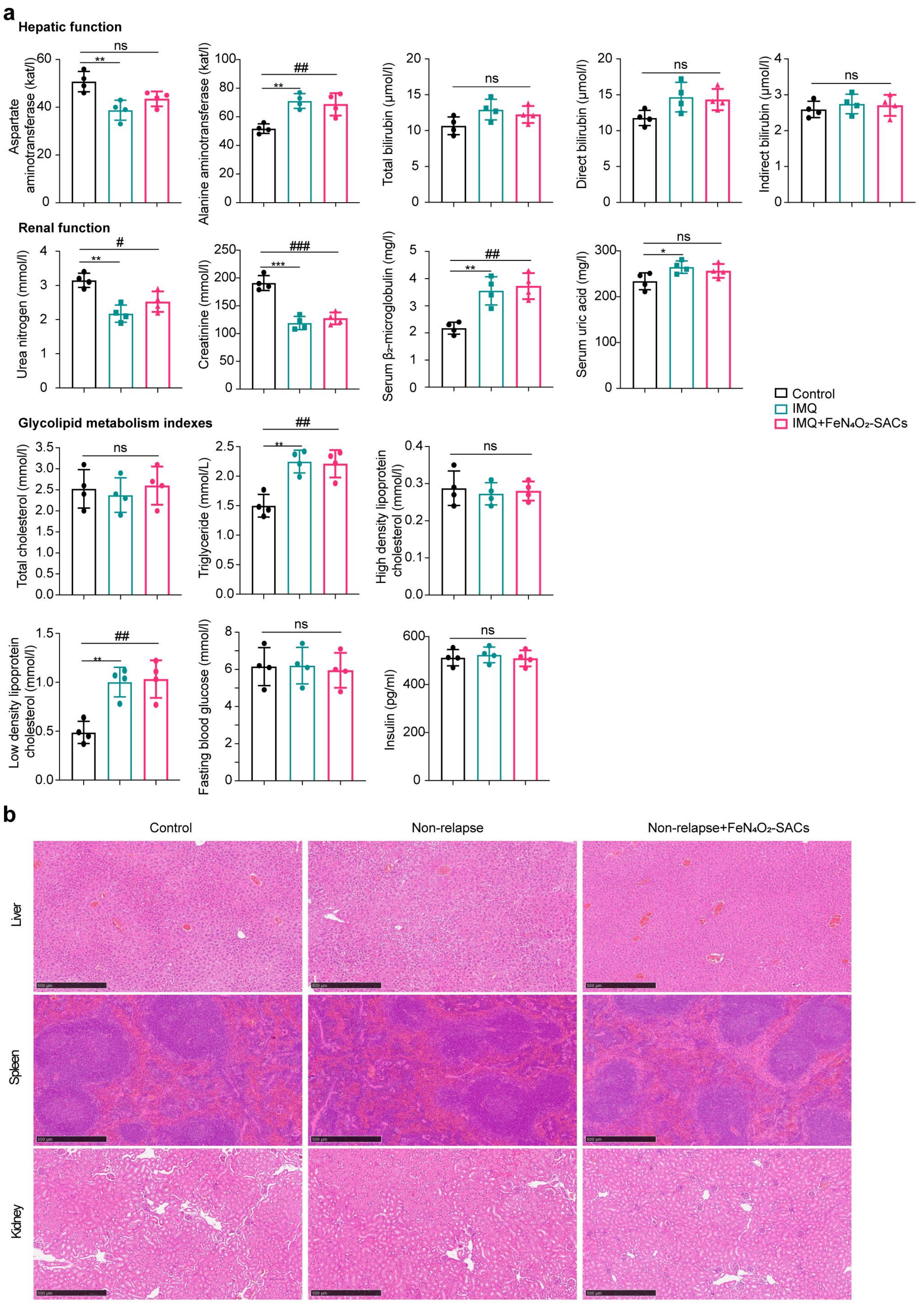

Supplementary Fig. 30. The organ toxicity detections of FeN_4O_2-SACs. a, The hepatic function

(alanine aminotransferase, aspartate aminotransferase, total bilirubin, direct bilirubin, and indirect bilirubin), renal function (urea nitrogen, creatinine, serum β2-microglobulin, and serum uric acid), and glycolipid metabolism indexes (total cholesterol, triglyceride, high density lipoprotein cholesterol, low density lipoprotein cholesterol, fasting blood glucose, and insulin) detected by ELISA, n = 4 samples/group and data are presented as mean ± SD. Statistical significance was calculated via Student's *t*-test. **b**, The histopathology of liver, spleen and kidney before and after treatments. n = 3 samples/group. $^{***}p<0.001$, $^{**}p<0.001$, $^{*}p<0.001$; $^{###}p<0.001$, $^{##}p<0.01$, $^{#}p<0.05$; ns means no significance. Source data are provided as a Source Data file.

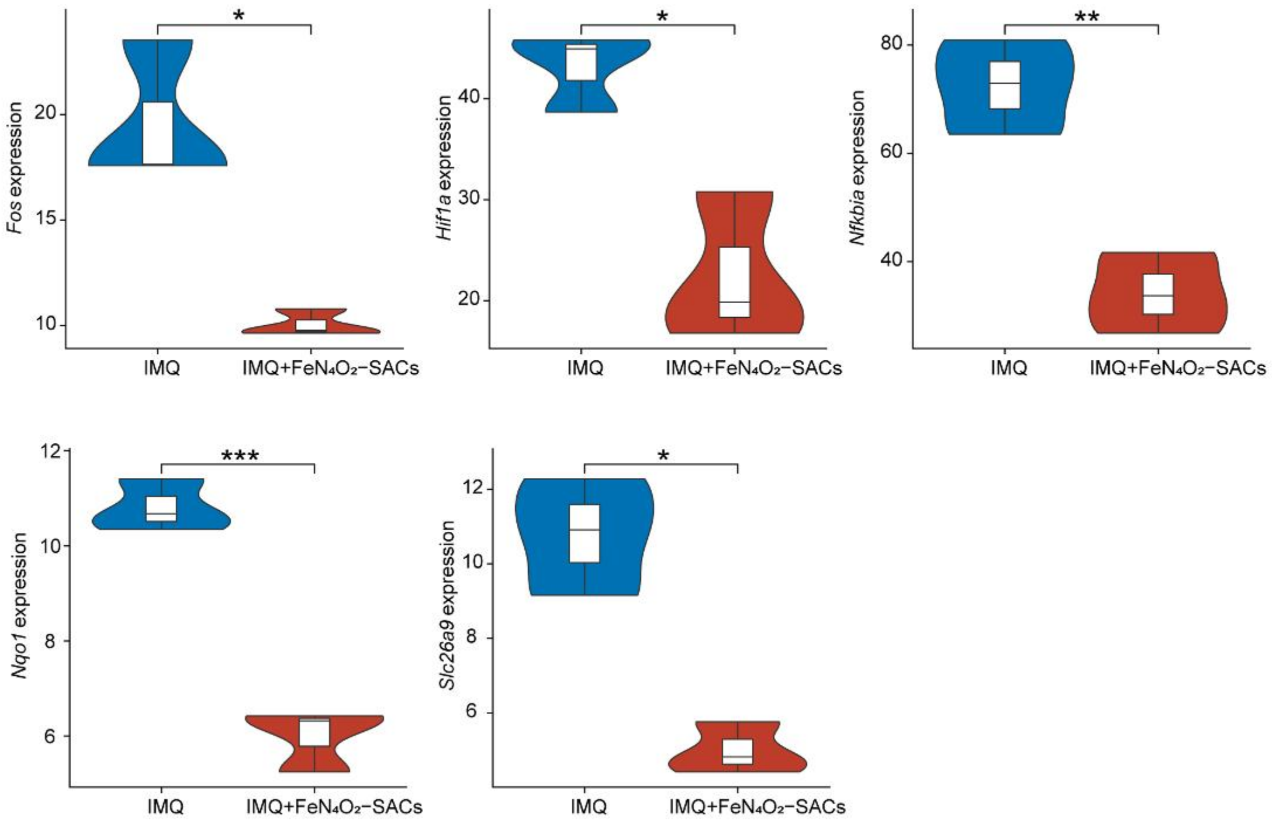

Supplementary Fig. 31. The core genes downregulated in the IMQ+FeN_4O_2-SACs group. ****p*<0.001, ***p*<0.01, **p*<0.05 versus the IMQ group. Statistical significance was calculated via Student's *t*-test.

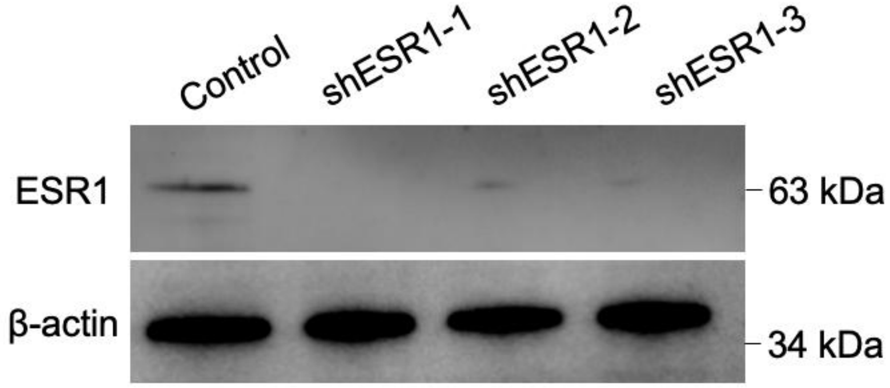

Supplementary Fig. 32. Western blotting analyses for the knock-down efficiency of ESR1 by shRNA, in which shESR1-1 was chosen for the following experiments. The samples derive from the same experiment and that gels/blots were processed in parallel.

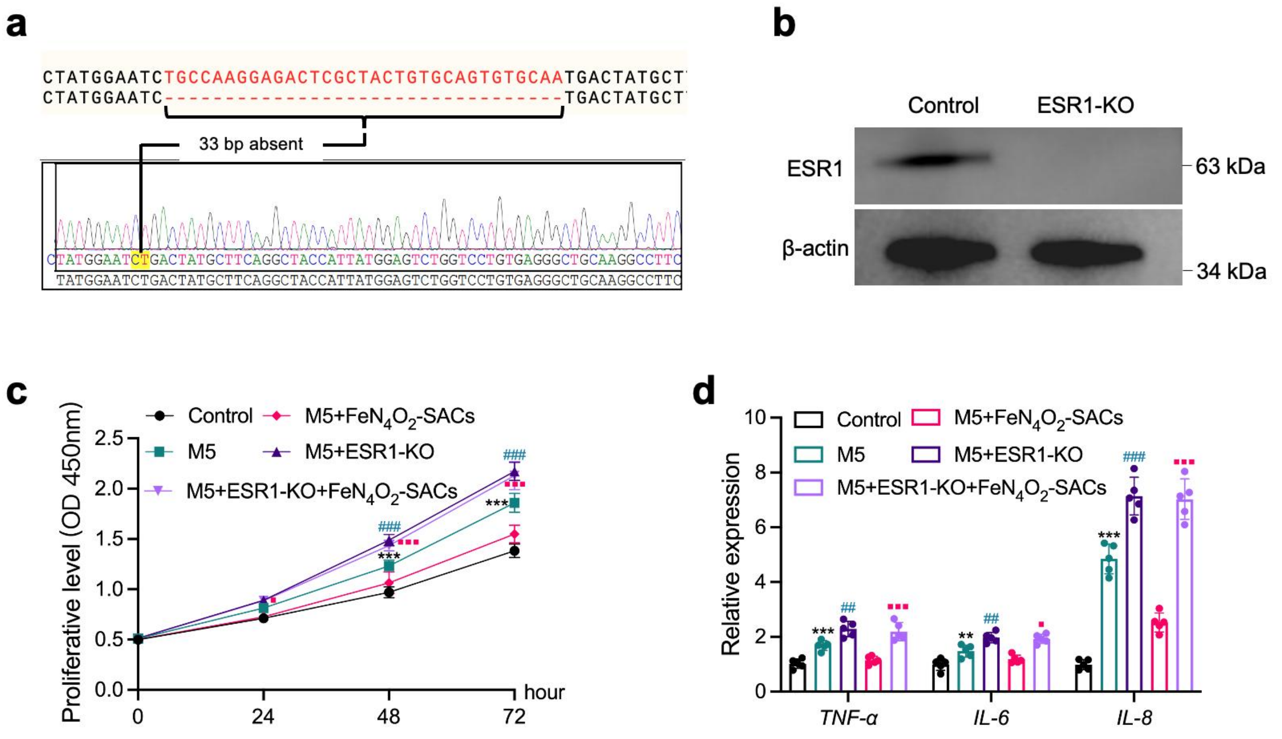

Supplementary Fig. 33. FeN_4O_2-SACs inhibit the proliferation and inflammation of HaCaT cells through ESR1. a,**b**, Knockout (KO) validation of ESR1 in HaCaT cells by sequencing (**a**) and western blotting (**b**). The samples derive from the same experiment and that gels/blots were processed in parallel. **c**, CCK-8 assay showing the cell proliferative viabilities regulated by ESR1-KO and the effect of FeN_4O_2-SACs. n = 3 independent experiments and data are presented as mean ± SD. **d**, RT-qPCR results for the inflammatory factors *TNF-α*, *IL-6*, and *IL-8* at 24 hours. n = 5 independent experiments and data are presented as mean ± SD. ****p*<0.001, ***p*<0.01 versus the Control group; ###*p*<0.001, ##*p*<0.01 versus the M5 group; ■■■*p*<0.001, ■*p*<0.05 versus the M5+ FeN_4O_2-SACs group. Statistical significance was calculated via two-way ANOVA (**c**), one-way ANOVA (**d**). Source data are provided as a Source Data file.

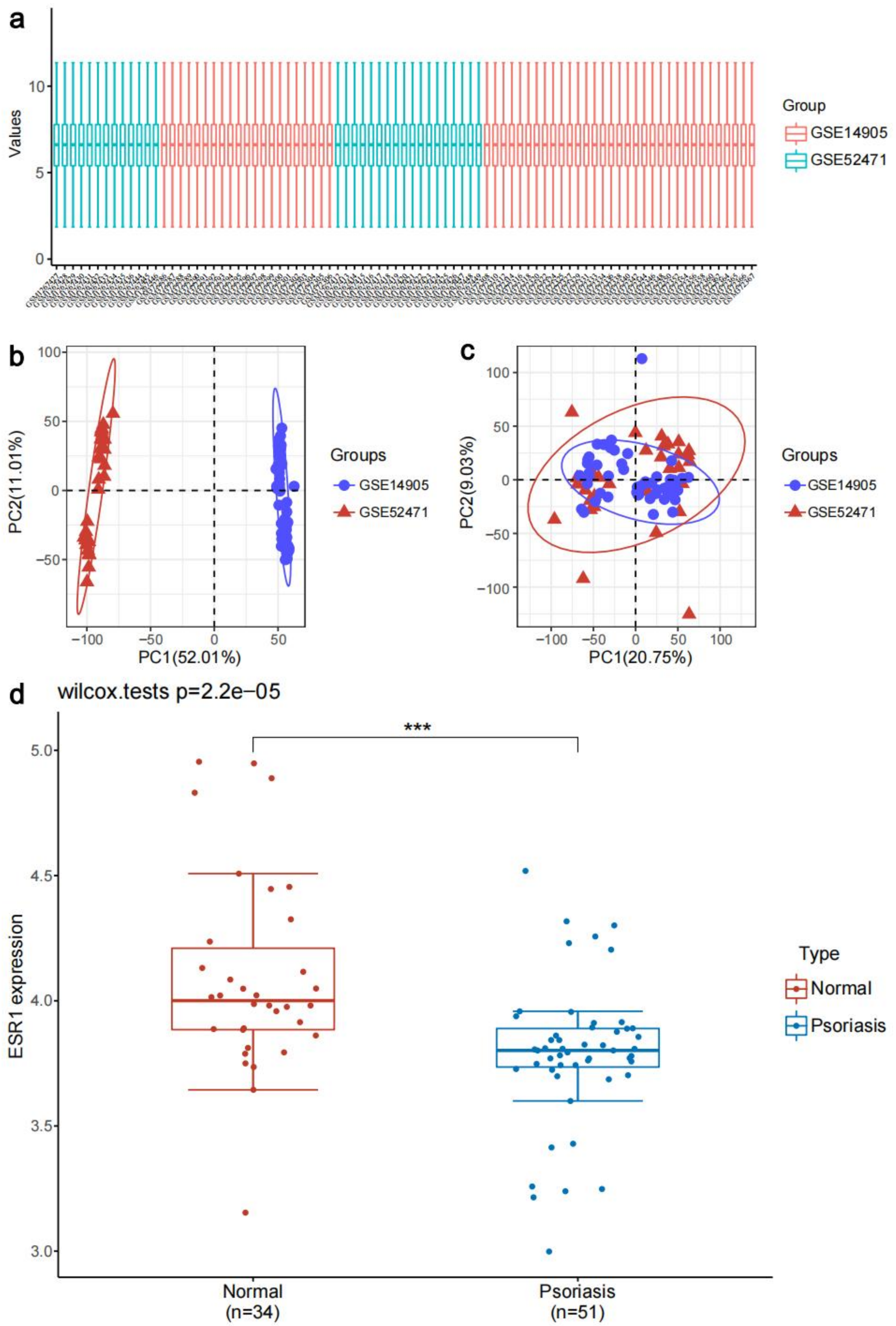

Supplementary Fig. 34. a, Box diagram after data standardization. Different colors represent different data sets. Rows indicate samples, and columns denote gene expression values in samples. **b**,

PCA results before batch removal of three datasets. Different colors represent different datasets. The two datasets are separated without any intersection. **c**, PCA result chart after removing the batch. The two datasets can be used as a batch of data for subsequent analysis as they are intersected together. **d**, *ESR1* expression in normal and psoriatic samples of two datasets. ***$p<0.001$. Statistical significance was calculated via Student's *t*-test.

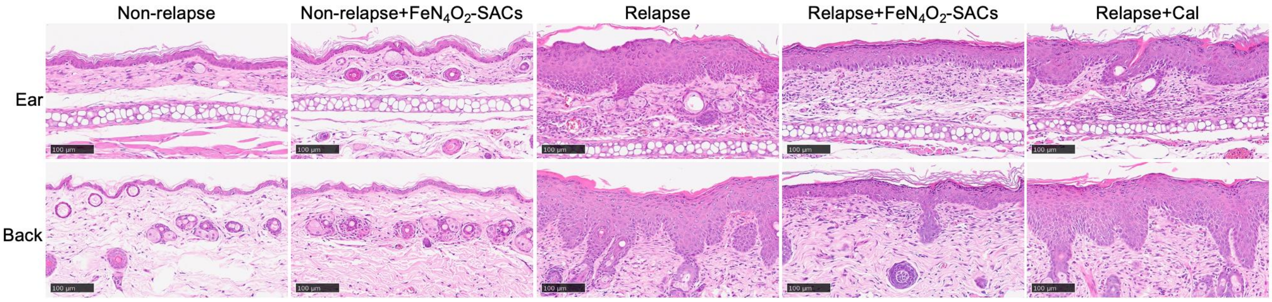

Supplementary Fig. 35. Histological staining of relapse models (ear and back). Scale bar = 100 μm, n = 4 samples/group.

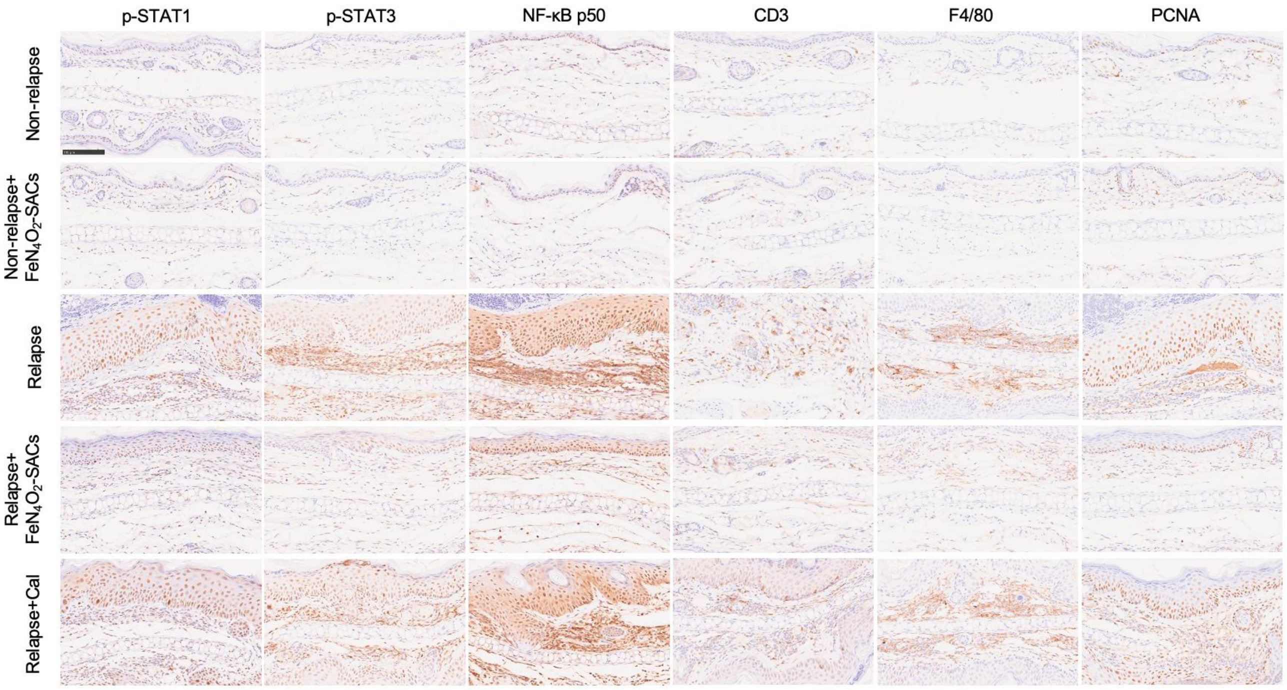

Supplementary Fig. 36. IHC staining of p-STAT1, p-STAT3, NF-κB p50, CD3, F4/80 and PCNA in lesional ear skin tissues from different groups. Scale bar = 100 μm, n = 4 samples/group.

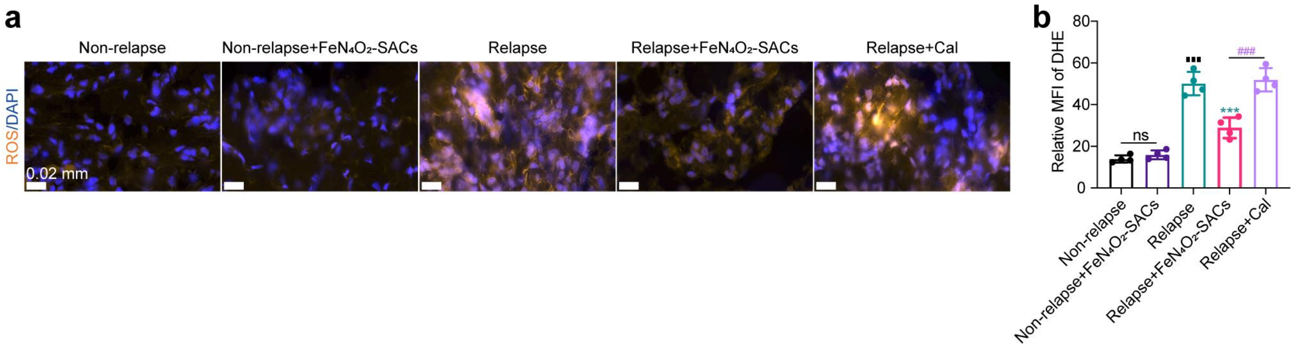

Supplementary Fig. 37. a,**b**, The ROS level of relapsed or non-relapsed mouse ear lesions analyzed using fluorescence probe DHE (Dihydroethidium, Filter block of spORANGE at EX532-554/EM576-596 nm) (**a**) and the quantification (**b**) of relative mean fluorescence intensity (MFI), orange color indicates the ROS-positive, n = 4 samples/group and data are presented as mean ± SD. $^{***}p$<0.001 versus the Relapse group; $^{\#\#\#}p$<0.001 versus the Relapse+FeN_4O_2-SACs group; ■■■p<0.001 versus the Non-relapse group; ns means no significance. Statistical significance was calculated via one-way ANOVA. Source data are provided as a Source Data file.

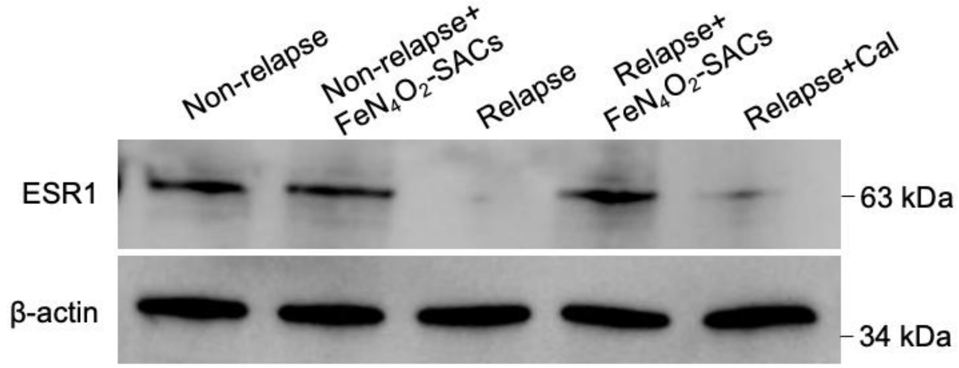

Supplementary Fig. 38. The protein expressions of ESR1 at the ear skin lesions of different groups. The samples derive from the same experiment and that gels/blots were processed in parallel.

3. Supplementary Tables

Supplementary Table 1. Fitted Mössbauer parameters. Isomer shift (IS), quadrupole splitting (QS), full width at half maximun (FWHM) and relative spectral area (A) of each species.

Sample	Fe species	IS (mm/s)	QS (mm/s)	FWHM (mm/s)	A (%)	Assinments
FeN_4O_2-SACs	D1	0.395	0.43	0.10	25.6	$Fe^{2+}N_4$
	D3	0.419	1.18	0.23	74.4	Fe^{3+} porphyrin

Supplementary Table 2. EXAFS fitting parameters at the Fe K-edge（S_0^2=0.90）

Sample	Path	C.N.	R (Å)	$\sigma^2\times10^3$ (Å^2)	ΔE (eV)	R factor
Fe foil	Fe-Fe	8*	2.47±0.01	4.0±1.7	6.0±2.6	0.002
	Fe-Fe	6*	2.84±0.02	4.8±3.4	4.7±4.1	
FeN_4O_2-SACs	Fe-O/N	6.4±1.6	1.99±0.02	16.6±3.6	0.2±2.5	0.010
	Fe-C	7.9±5.0	2.90±0.03	8.5±5.7	-9.1±4.6	

C.N: coordination numbers; *R*: bond distance; σ^2: Debye-Waller factors; Δ*E*: the inner potential correction. *R* factor: goodness of fit. * fitting with fixed parameter.

Supplementary Table 3. Comparison of the enzymatic catalytic activities of FeN_4O_2-SACs with commercial antioxidant single-atom catalysts.

groups	SOD (units/mg)	CAT (units/mg)	APX (units/mg)
Fe in FeN_4O_2-SACs	21779.51	20828.59	354833.89
Ce in CeO_2	34.99	9.82	27.73
Mn in Mn_3O_4	63.61	44.22	16.85
Co in Co-SACs	2913.98	3830.22	77186.15
Cu in Cu-SACs	3792.52	2593.10	32619.05
Zn in Zn-SACs	57.67	88.12	240.15

Supplementary Table 4. Comparison of the enzymatic catalytic activities of FeN_4O_2-SACs with other reported SACs antioxidant nanoenzyme.

Materials	ICP	SOD (units/mg)	CAT (units/mg)	APX (units/mg)
Fe in FeN_4O_2-SACs	0.43%	21779.51	20828.59	354833.89
Ir in Ir NC SAzymes[5]	0.6%	-	9728.33	-
Fe in Fe-SANzyme[6]	0.45%	-	11697.78	-
Cu in Cu SAs/CN[7]	0.18%	-	-	256444.44
Rh in RhN_4 nanozymes[8]	0.19%	574.29	50306.09	-
V in VN_4 nanozymes[8]	0.22%	803.92	12176.47	-

Supplementary Table 5. Primers for RT-qPCR

Gene	Forward primer	Reverse primer
Human *TNF-α*	CTCTTCTGCCTGCTGCACTTTG	ATGGGCTACAGGCTTGTCACTC
Human *IL-6*	AGACAGCCACTCACCTCTTCAG	TTCTGCCAGTGCCTCTTTGCTG
Human *IL-8*	GAGAGTGATTGAGAGTGGACCAC	CACAACCCTCTGCACCCAGTTT
Human *FOS*	CAGACTACGAGGCGTCATCC	TCTGCGGGTGAGTGGTAGTA
Human *HIF1A*	GATCACCCTCTTCGTCGCTT	CCTCCATGGTGAATCGGTCC
Human *NFKBIA*	GAAGTGATCCGCCAGGTGAA	CTCACAGGCAAGGTGTAGGG
Human *NQO1*	CTGAAGGACCCTGCGAACTT	GAACACTCGCTCAAACCAGC
Human *SLC26A9*	CAGATATGAGCCAGCCCAGG	TGGGTATGTCCGGTCCTTCT
Human *β-ACTIN*	GTCATTCCAAATATGAGATGCGT	TGTGGACTTGGGAGAGGACT
Mouse *Il-17a*	GGAGAGCTTCATCTGTGTCTCTG	TTGAGGGATGATCGCTGCTG
Mouse *Tnf-α*	GGTGCCTATGTCTCAGCCTCTT	GCCATAGAACTGATGAGAGGGAG
Mouse *Il-12*	AGAGAGATTCACAGGGCACAG	CATCAGCATCAGTGCCACAC
Mouse *Il-23*	ACCAGCGGGACATATGAATCT	AGACCTTGGCGGATCCTTTG
Mouse *Il-10*	CAGTGGAGCAGGTGAAGAGT	TGGAGTCCAGCAGACTCAATAC
Mouse *Esr1*	AAGACGCTCTTGAACCAGCA	AGGCTTTGGTGTGAAGGGTC
Mouse *Fos*	TACTACCATTCCCCAGCCGA	GCTGTCACCGTGGGGATAAA
Mouse *Hif1a*	CTTGACAAGCTAGCCGGAGG	TCGACGTTCAGAACTCATCCT
Mouse *Nfkbia*	TGGCCAGTGTAGCAGTCTTG	AGGGGGAGTAGCCTTGGTAG
Mouse *Nqo1*	TAGCCTGTAGCCAGCCCTAA	GCCTCCTTCATGGCGTAGTT
Mouse *Slc26a9*	GAGCCCTTTCACACACCTCA	GCTCTGTCTACCACGTAGCG
Mouse *β-actin*	GAGCGCAAGTACTCTGTGTG	GGTGTAAAACGCAGCTCAGTAA

4. Supplementary Discussion

Analysis of XRD, FT-IR spectroscopy and Raman spectroscopy

The XRD pattern of FeN_4O_2-SACs only exhibits a broadened peak of graphitic carbon, demonstrating its poor crystallinity and homogeneous Fe single atoms without Fe-based crystalline nanoparticles (Supplementary Fig. 7). FTIR spectra show that both Zn-MOF and Fe-MOF exhibit a characteristic peak of imidazole rings at 800–1500 cm^{-1}. After pyrolysis, the characteristic peaks disappeared and the two absorption bands at 1300 and 1590 cm^{-1} for NC and FeN_4O_2-SACs can be assigned to C-N and C=N stretching vibrations, respectively (Supplementary Fig. 8). Therefore, it could be inferred that FeN_4O_2-SACs possess a carbon structure similar to that of NC, which might be related to the low level of Fe doping. The result is consistent well with the Raman characterization. As described in Supplementary Fig. 9, both FeN_4O_2-SACs and NC display two peaks at 1348 and 1580 cm^{-1}, which correspond to the D (disorder) and G (graphite) bands of carbon, respectively[9,10]. The I_D/I_G ratio did not change much after Fe doping, indicating that FeN_4O_2-SACs and NC have similar structural defects. Meanwhile, no peaks of impurities such as Fe and Fe_3O_4 crystals were detected in the Raman spectra.

5. Supplementary References

1. Kuai, L. *et al.* PD-L1 Triggered by Binding eIF3I Contributes to the Amelioration of Diabetes-Associated Wound Healing Defects by Regulating IRS4. *J. Invest. Dermatol.* **142**, 220-231.e228 (2022).
2. Polakova, A. *et al.* Isolation of skin-derived lymphocytes from human skin and murine tissues: A rapid and epitope-preserving approach. *Exp. Dermatol.* **31**, E67-E67 (2022).
3. Bader, G. D. & Hogue, C. W. An automated method for finding molecular complexes in large protein interaction networks. *BMC Bioinform.* **4**, 2 (2003).
4. Tang, Y., Li, M., Wang, J., Pan, Y. & Wu, F. X. CytoNCA: a cytoscape plugin for centrality analysis and evaluation of protein interaction networks. *Biosystems* **127**, 67-72 (2015).
5. Wang, Z. *et al.* Single-Atom Catalysts with Ultrahigh Catalase-Like Activity Through Electron Filling and Orbital Energy Regulation. *Adv. Funct. Mater.* **33**, 2209560 (2023).
6. Zhang, R. *et al.* Edge-Site Engineering of Defective Fe-N4 Nanozymes with Boosted Catalase-Like Performance for Retinal Vasculopathies. *Adv. Mater.* **34**, e2205324 (2022).
7. Chen, Y. *et al.* Atomically Dispersed Cu Nanozyme with Intensive Ascorbate Peroxidase Mimic Activity Capable of Alleviating ROS-Mediated Oxidation Damage. *Adv. Sci.* **9**, 2103977 (2022).
8. Zhang, S. F. *et al.* Single-atom nanozymes catalytically surpassing naturally occurring enzymes as sustained stitching for brain trauma. *Nat. Commun.* **13**, 4744 (2022).
9. Lu, X. *et al.* Bioinspired Copper Single-Atom Catalysts for Tumor Parallel Catalytic Therapy. *Adv. Mater.*, 2002246 (2020).
10. Liu, W. *et al.* Discriminating Catalytically Active FeN_x Species of Atomically Dispersed Fe–N–C Catalyst for Selective Oxidation of the C–H Bond. *J. Am. Chem. Soc.* **139**, 10790-10798 (2017).
