# Supplementary material for: Single-atom catalysts-based catalytic ROS clearance for efficient psoriasis treatment and relapse prevention via restoring ESR1
Source: Nat Commun. 2023 Oct 25;14:6767. doi: 10.1038/s41467-023-42477-y (PMC10600197; doi:10.1038/s41467-023-42477-y)
Supplement: Supplementary file 2 — Reporting Summary [file 41467_2023_42477_MOESM2_ESM.pdf]

nature portfolio

Corresponding author(s): Jianlin Shi

Last updated by author(s): Sep 30, 2023

Reporting Summary

Nature Portfolio wishes to improve the reproducibility of the work that we publish. This form provides structure for consistency and transparency in reporting. For further information on Nature Portfolio policies, see our Editorial Policies and the Editorial Policy Checklist.

Statistics

For all statistical analyses, confirm that the following items are present in the figure legend, table legend, main text, or Methods section.

n/a	Confirmed	
☐	☒	The exact sample size (*n*) for each experimental group/condition, given as a discrete number and unit of measurement
☐	☒	A statement on whether measurements were taken from distinct samples or whether the same sample was measured repeatedly
☐	☒	The statistical test(s) used AND whether they are one- or two-sided<br>*Only common tests should be described solely by name; describe more complex techniques in the Methods section.*
☒	☐	A description of all covariates tested
☒	☐	A description of any assumptions or corrections, such as tests of normality and adjustment for multiple comparisons
☐	☒	A full description of the statistical parameters including central tendency (e.g. means) or other basic estimates (e.g. regression coefficient) AND variation (e.g. standard deviation) or associated estimates of uncertainty (e.g. confidence intervals)
☐	☒	For null hypothesis testing, the test statistic (e.g. *F*, *t*, *r*) with confidence intervals, effect sizes, degrees of freedom and *P* value noted<br>*Give P values as exact values whenever suitable.*
☒	☐	For Bayesian analysis, information on the choice of priors and Markov chain Monte Carlo settings
☒	☐	For hierarchical and complex designs, identification of the appropriate level for tests and full reporting of outcomes
☒	☐	Estimates of effect sizes (e.g. Cohen's *d*, Pearson's *r*), indicating how they were calculated

Our web collection on statistics for biologists contains articles on many of the points above.

Software and code

Policy information about availability of computer code

Data collection	Microsoft Excel (version 2019); Flow cytometry (BD LSR Fortessa); Slide Viewer 2.6.0; NDP. view 2 for Mac
Data analysis	Microsoft Excel (version 2019); Origin Pro (version 2023); Graphpad Prism 8; FlowJo (version 10.8.1).

For manuscripts utilizing custom algorithms or software that are central to the research but not yet described in published literature, software must be made available to editors and reviewers. We strongly encourage code deposition in a community repository (e.g. GitHub). See the Nature Portfolio guidelines for submitting code & software for further information.

Data

Policy information about availability of data

All manuscripts must include a data availability statement. This statement should provide the following information, where applicable:

- Accession codes, unique identifiers, or web links for publicly available datasets
- A description of any restrictions on data availability
- For clinical datasets or third party data, please ensure that the statement adheres to our policy

All the data supporting the findings of this study are available within the article, source data, and its supplementary information files. Source data are provided as a Source Data file. The raw data of RNA-sequencing has been deposited in the Entrez Molecular Sequence Database System under BioProject accession number of PRJNA909970 (https://www.ncbi.nlm.nih.gov/bioproject/PRJNA909970/). This study uses publicly available data from the Protein Data Bank (PDB) under accession codes: 1DGF (https://doi.org/10.2210/pdb1dgf/pdb), 1V0H (https://doi.org/10.2210/pdb1V0H/pdb), and 1AVM (https://doi.org/10.2210/pdb1AVM/pdb).

Research involving human participants, their data, or biological material

Policy information about studies with human participants or human data. See also policy information about sex, gender (identity/presentation), and sexual orientation and race, ethnicity and racism.

Reporting on sex and gender	Sex and gender were not considered in study design.
Reporting on race, ethnicity, or other socially relevant groupings	*Please specify the socially constructed or socially relevant categorization variable(s) used in your manuscript and explain why they were used. Please note that such variables should not be used as proxies for other socially constructed/relevant variables (for example, race or ethnicity should not be used as a proxy for socioeconomic status).*<br>*Provide clear definitions of the relevant terms used, how they were provided (by the participants/respondents, the researchers, or third parties), and the method(s) used to classify people into the different categories (e.g. self-report, census or administrative data, social media data, etc.)*<br>*Please provide details about how you controlled for confounding variables in your analyses.*
Population characteristics	Healthy individuals and psoriasis patients at both incipient and relapse stages consistent with the clinical diagnosis aged 18-65 were included. Psoriasis and relapse samples were collected from the lesional skin of patients, and normal skin samples were collected from excess skin during cosmetic surgeries.
Recruitment	Skin samples were collected and used for immunohistochemistry (IHC) staining.
Ethics oversight	Ethics Committee of Yueyang Hospital affiliated to Shanghai University of Traditional Chinese Medicine (no. 2019-29).

Note that full information on the approval of the study protocol must also be provided in the manuscript.

Field-specific reporting

Please select the one below that is the best fit for your research. If you are not sure, read the appropriate sections before making your selection.

☒ Life sciences ☐ Behavioural & social sciences ☐ Ecological, evolutionary & environmental sciences

For a reference copy of the document with all sections, see nature.com/documents/nr-reporting-summary-flat.pdf

Life sciences study design

All studies must disclose on these points even when the disclosure is negative.

Sample size	No statistical methods were used to predetermine the sample sizes. The group sizes (at least three animals in each group) represent the minimal number of animals needed to reach statistical significance ($p < 0.05$) between experimental groups.
Data exclusions	No data were excluded.
Replication	All experiments were repeated at least three times with similar results.
Randomization	Cells or mice were randomly assigned to different groups before treatments.
Blinding	CCK-8 assays, flow cytometry analysis, RT-PCR and alizarin red S staining were conducted by experienced operators who were blinded to the treatment groups.<br>In histological assessments, tissues were analyzed by an experienced investigator who was blinded as to treatment-group assignment.<br>For other experiments, investigators were also blinded to the group allocation during data analysis.

Reporting for specific materials, systems and methods

We require information from authors about some types of materials, experimental systems and methods used in many studies. Here, indicate whether each material, system or method listed is relevant to your study. If you are not sure if a list item applies to your research, read the appropriate section before selecting a response.

Materials & experimental systems

n/a	Involved in the study
☐	☒ Antibodies
☐	☒ Eukaryotic cell lines
☒	☐ Palaeontology and archaeology
☐	☒ Animals and other organisms
☐	☒ Clinical data
☐	☒ Dual use research of concern
☒	☐ Plants

Methods

n/a	Involved in the study
☒	☐ ChIP-seq
☐	☒ Flow cytometry
☒	☐ MRI-based neuroimaging

Antibodies

Antibodies used

1. CD3 antibody, ab16669, Abcam (https://www.abcam.cn/products/primary-antibodies/cd3-antibody-sp7-ab16669.html);
2. F4/80 antibody, #70076, Cell Signaling (https://www.cellsignal.cn/products/primary-antibodies/f4-80-d2s9r-xp-rabbit-mab/70076?site-search-type=Products&N=4294956287&Ntt=%2370076&fromPage=plp&_requestid=1179574);
3. PCNA antibody, NA03-200UG, Sigma (https://www.sigmaaldrich.cn/CN/zh/search/na03-200ug focus=products&page=1&perpage=30&sort=relevance&term=na03-200ug&type=product);
4. ESR1 antibody, ab32063, Abcam (https://www.abcam.cn/products/primary-antibodies/estrogen-receptor-alpha-antibody-e115-chip-grade-ab32063.html);
5. ESR1 antibody, PA5-16440, Invitrogen (https://www.thermofisher.cn/cn/zh/antibody/product/Estrogen-Receptor-alpha-Antibody-Polyclonal/PA5-16440).
6.p-STAT1:https://www.abcam.cn/products/primary-antibodies/stat1-phospho-s727-antibody-epr3146-ab109461.html
7.p-STAT3 ：https://www.abcam.cn/products/primary-antibodies/stat3-phospho-y705-antibody-ep2147y-ab76315.html
8.NF kappa B p50:https://www.abcam.cn/products/primary-antibodies/nfkb-p105--p50-antibody-e381-ab32360.html
9.CD103: https://www.abcam.cn/products/primary-antibodies/cd103-antibody-epr22590-27-ab224202.html
10.CD45-BV421(30-F11, Biolegend, 103134), CD3-FITC (17A2, Biolegend,100204), CD4-PerCP (RM405, Biolegend, 100538), CD8-PE (53-6.7, Biolegend, 100708), F4/80-APC (BM8, Invitrogen, 17-4801-82), CD11b-BV785 (M1/70, Biolegend, 101243), Viability-APC-Cy7 (Invitrogen, 65-0865-14), and CD103-BV510 (2E7, Biolegend, 121423)

Validation

All antibodies were verified by the supplier, and each lot has been quality-tested. These antibodies are used without additional validation.

Eukaryotic cell lines

Policy information about cell lines and Sex and Gender in Research

Cell line source(s)	The human immortal keratinocyte line HaCaT were obtained from Cell Lines Service, Eppelheim, 300493. The normal human epidermis keratinocytes (NHEK) were obtained from BeNa Culture Collection, China, 340593.
Authentication	Cell lines were authenticated by Cell Lines Service using specific methods (Karyotyping, DNA barcoding, PCR assays with species-specific primers, etc.)
Mycoplasma contamination	Cell lines tested negative for mycoplasma.
Commonly misidentified lines (See ICLAC register)	No commonly misidentified cell lines were used.

Animals and other research organisms

Policy information about studies involving animals; ARRIVE guidelines recommended for reporting animal research, and Sex and Gender in Research

Laboratory animals	Male BALB/c mice aged 6-8 weeks were provided by Shanghai SLAC Laboratory Animal Co., Ltd., (no. 20220004020279, SYXK (Hu) 2018-0040).
Wild animals	No wild animals were included.
Reporting on sex	To avoid the effect of sex hormones secreted by female mice on the results, only male BALB/c mice were included in the study.
Field-collected samples	The study did not involve samples collected from the field.
Ethics oversight	All the animal procedures were approved by Ethics Committee of Yueyang Hospital affiliated to Shanghai University of Traditional Chinese Medicine (no. YYLAC-2021-107-6, YYLAC-2022-160-3).

Note that full information on the approval of the study protocol must also be provided in the manuscript.

Clinical data

Policy information about clinical studies
All manuscripts should comply with the ICMJE guidelines for publication of clinical research and a completed CONSORT checklist must be included with all submissions.

Clinical trial registration	Ethics No. 2019-029
Study protocol	No study protocol is available online, because no registered clinical trial is involved. This study collected human skin biospecimens with the approval of Ethics Committee of Yueyang Hospital affiliated to Shanghai University of Traditional Chinese Medicine (no. 2019-29).
Data collection	Skin samples were collected from the patients who were undergoing a surgery with informed consent signed. Then the skin tissues were immediately kept in 4% formalin for 48h and immunohistochemistry (IHC) staining were performed. The data quantification was collected with Image J for Mac (version 1.53).
Outcomes	Live cells were selected by FSC and SCC analysis. Green fluorescence of DCF in cells was detected by FITC channel.

Flow Cytometry

Plots

Confirm that:

- [x] The axis labels state the marker and fluorochrome used (e.g. CD4-FITC).
- [x] The axis scales are clearly visible. Include numbers along axes only for bottom left plot of group (a 'group' is an analysis of identical markers).
- [x] All plots are contour plots with outliers or pseudocolor plots.
- [x] A numerical value for number of cells or percentage (with statistics) is provided.

Methodology

Sample preparation	The treated cells were washed with PBS, collected by trypsinization iwithout (EDTA), resuspended in cold PBS. Afterwards, obtained cells were stained with specific dyes according to the manufacturer's instruction and analyzed by flow cytometry.
Instrument	BD LSR Fortessa
Software	FlowJo (version 10.8.1)
Cell population abundance	No cell sorting was performed.
Gating strategy	Live cells were selected by FSC and SCC analysis. Green fluorescence of DCF in cells was detected by FITC channel.

- [x] Tick this box to confirm that a figure exemplifying the gating strategy is provided in the Supplementary Information.
